# Cost−effectiveness of pasireotide as second−line therapy for adult acromegaly patients from the perspective of the Brazilian Unified Health System

**DOI:** 10.3389/fendo.2026.1771580

**Published:** 2026-08-05

**Authors:** Vania dos Santos Nunes Nogueira, Grzegorz Binowski, Małgorzata Bronikowska, Fabian Schmidt, Beatrice Gueron, Helder Paiva, Sika Dédé Kossi

**Affiliations:** 1 Department of Internal Medicine, Universidade Estadual Paulista (UNESP), Faculdade de Medicina, Botucatu, Botucatu, São Paulo, Brazil; 2 MAHTA Intl., Warsaw, Poland; 3 Recordati Rare Diseases, Puteaux, France; 4 Recordati Rare Diseases, São Paulo, Brazil

**Keywords:** acromegaly, cost-effectiveness, first-generation somatostatin receptor ligand, incremental cost-effectiveness ratio, pasireotide, pegvisomant, quality-adjusted life years

## Abstract

**Background:**

Acromegaly is a rare, chronic disease caused by excessive growth hormone (GH) production due to pituitary adenoma. The disease severely impacts patients’ health-related quality of life and mortality while imposing a substantial economic burden. Resistance to first-line medical treatment necessitates effective second-line therapies, including pasireotide long-acting release (PAS LAR) and pegvisomant (PEG).

**Objective:**

To evaluate the cost-effectiveness of PAS LAR versus PEG (monotherapy or combined with FGSRLs) and versus FGSRLs alone, as second-line medical treatments for adults with acromegaly who are not adequately controlled on FGSRLs, from the perspective of the Brazilian Unified Health System (SUS).

**Methods:**

A hybrid decision-tree and Markov model were developed to evaluate lifetime health and economic outcomes of assessed drugs. To inform the model, health outcomes data were sourced from clinical trials, real-world evidence, systematic reviews, and meta-analyses. Costs were extracted from the official Brazilian tariffs database. Comparators included the FGSRLs, lanreotide (LAN) and octreotide (OCT), and PEG, as monotherapy and in combination with FGSRLs. Deterministic and probabilistic sensitivity analysis were performed to assess the impact of uncertainty on results. The incremental cost-effectiveness ratio (ICER) was the primary analysis outcome.

**Results:**

PAS LAR was dominant versus PEG monotherapy and in combination with LAN, generating similar health benefits (an additional 0.17 and 0.10 QALYs, respectively) and lower therapy costs (savings of -R$538,945 [-US$100,675] and -R$782,542 [-US$146,179]). In the comparison with PEG combined with OCT, PAS LAR was less costly (-R$979,624 [-US$182,994]) and marginally less effective (-0.04 QALYs). Compared to FGSRLs, PAS LAR demonstrated improved health outcomes (QALYs) and increased cost. The ICERs were R$161,411 (US$30,152)/QALY and R$168,538 (US$31,483)/QALY versus OCT and LAN, respectively. Scenario analyses confirmed robustness across variations in key model inputs that have the highest impact on cost-effectiveness results.

**Conclusions:**

The findings showed that in the Brazilian SUS context, PAS LAR is cost-effective compared to PEG monotherapy and PEG + LAN, and cost-saving versus PEG + OCT. Compared to FGSRLs, PAS LAR provided better outcomes with higher costs. With limited options for second-line medical treatment in Brazil, the reimbursement of PAS LAR in acromegaly can be considered a cost-effective strategy for acromegaly patients.

## 1 Introduction

Acromegaly is a rare, chronic endocrine disorder caused by excessive production of growth hormone (GH) and insulin-like growth factor 1 (IGF-1), often resulting from benign pituitary adenomas (1, 2). The global prevalence of acromegaly ranges from 1 in 7,500 to 1 in 35,800 individuals, with annual incidence rates estimated at approximately 1 in 91,000 to 1 in 526,000 (3–5). Severe complications involving the cardiovascular, respiratory, and metabolic systems are associated with acromegaly and contribute to increased morbidity and a two- to threefold higher mortality risk compared to the general population (6, 7). In addition, acromegaly and its comorbidities have a strong negative impact on patients’ health-related quality of life (HRQoL), with impaired scores across all domains of generic and disease-specific questionnaires (8, 9).

The primary therapeutic goals in managing acromegaly include normalization of IGF-1 levels (within the age- and sex-adjusted normal limits), alleviation of symptoms, reduction of tumor size, and minimization of long-term complications (10, 11). Surgical resection is the treatment of choice in acromegaly, with remission rates ranging from 40% to 80% (3, 4, 12). However, this indicates that surgery does not achieve disease control in a significant proportion of patients, many of whom experience persistent disease or recurrence. In such cases, or if surgery is not an option, medical therapy is used, and long-acting release formulations of first-generation somatostatin receptor ligands (FGSRL) (octreotide [OCT], lanreotide [LAN]) are recommended as first-line treatments (2, 13). Despite the proven efficacy of these agents, approximately 40% of patients fail to achieve adequate biochemical control or to maintain sustained remission (13, 14). This unmet need highlights the necessity for more effective second-line therapeutic options.

Available second-line medical treatment options are pasireotide long-acting release (PAS LAR), which acts on both GH and IGF-1, pegvisomant (PEG)-based regimens (monotherapy and combinations with an FGSRL), which target only IGF-1, and FGSRLs used at maximum doses, especially if PAS LAR and PEG are not available (2, 13, 15).

PAS LAR is a second-generation somatostatin receptor ligand with a broader receptor affinity profile (13). In contrast to FGSRLs, PAS LAR binds with high affinity to somatostatin receptors 2, 3, and 5, the three receptors expressed at high levels in most somatotroph adenomas, resulting in decreased GH secretion and a subsequent reduction in IGF-1 levels (16–18).

Clinical trials, including the PAOLA study (19), have demonstrated the ability of PAS LAR to improve biochemical control and quality of life in patients with persistent disease following FGSRL therapy. While PAS LAR is recommended as a second-line treatment option and approved in numerous markets worldwide (16, 17, 20), its widespread use in clinical practice remains influenced by cost-effectiveness considerations, especially in comparison to PEG.

This study aims to assess the cost-effectiveness of PAS LAR as a second-line treatment for adult patients with acromegaly in the Brazilian healthcare setting, compared with other approved second-line medical therapies. By developing a hybrid decision-tree and Markov model, the analysis will evaluate the health and economic outcomes associated with PAS LAR in comparison to alternative second-line pharmacological therapies. The findings are intended to provide robust evidence to guide healthcare decision-making, resource allocation optimization, and better health outcomes for adult patients with acromegaly for whom surgery is not an option or who remain inadequately controlled on FGSRL.

## 2 Methods

The cost-effectiveness analysis was conducted in accordance with the Brazilian Guideline for Economic Evaluation of Health Technologies, from the perspective of the Brazilian Unified Health System (Sistema Único de Saúde – SUS, public payer) (21), and is reported in compliance with the Consolidated Health Economic Evaluation Reporting Standards (CHEERS) checklist (22).

The study population consisted of adult patients with acromegaly for whom surgery was not an option or has failed to achieve adequate disease control and who were inadequately controlled (IGF-1 > upper limit normal [ULN]) on treatment with on-label FGSRL (OCT or LAN) regimen.

The intervention evaluated was PAS LAR administered at a starting dose of 40 mg by intramuscular injection every 28 days, with potential titration to 60 mg every 28 days according to clinical response, as per the approved prescribing information (17). The comparator was standard medical therapy with pharmacological treatments recommended by current international guidelines for acromegaly patients who remain uncontrolled with on-label FGSRL regimens. These included continuation of FGSRLs (OCT 40 mg or LAN 120 mg), switching to PEG (starting at 10 mg/day and titrated up to 30 mg/day according to clinical response), or a combination of these approaches.

While PAS LAR, OCT, and LAN are reimbursed by the Brazilian government (23), PEG is not formally reimbursed; however, its cost may be covered by the government on a case-by-case basis (23). Accordingly, adopting the perspective of the Brazilian public payer was deemed appropriate for the analysis.

The main outcomes evaluated were the cost per quality-adjusted life year (QALY), the incremental cost-effectiveness ratios (ICERs), and the incremental net monetary benefit (INMB).

### 2.1 Model structure and settings

**Figure 1:**
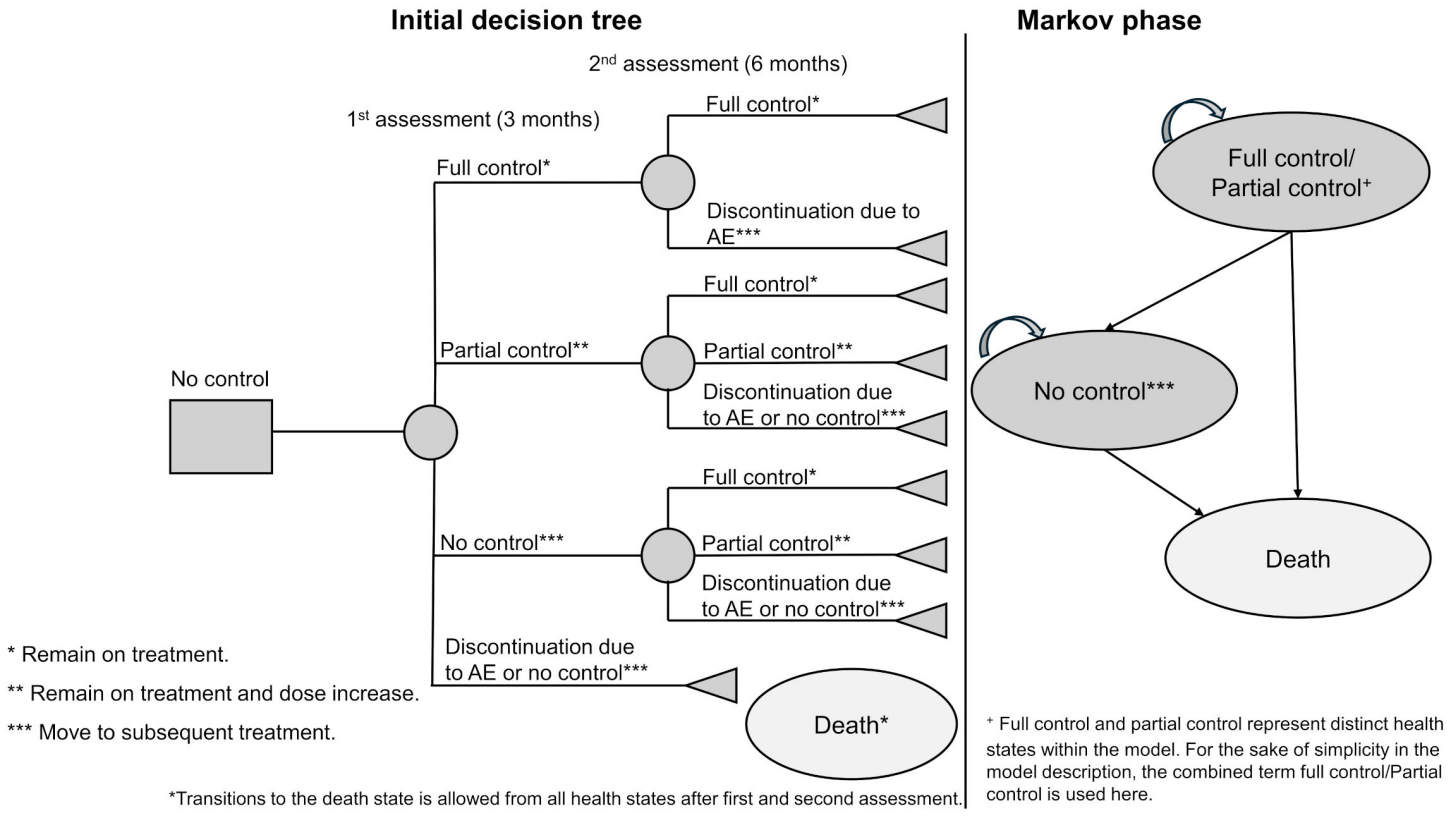
Model structure representing the pathway of patients for each treatment.

A hybrid model, combining a six-month decision-tree phase followed by a lifetime Markov model phase with six-month cycles, was developed in Microsoft Excel. The use of a Markov design was appropriate given that acromegaly is a chronic disease with recurrent health states. The model simulated treatment response (biochemical control) and failure (no control, loss of control, and discontinuation due to adverse events [AEs]) among adult patients with inadequately controlled acromegaly following treatment with on-label FGSRL regimens, in Brazil. A scheme of health states and possible transitions is presented in **Figure 1**. The efficacy data were sourced from clinical trials (19, 24, 25) identified through a systematic literature review (SLR) (details provided in **Supplementary Material 1**) and derived from an indirect treatment comparison (ITC) (details provided in **Supplementary Material 2**) and real-world evidence studies (26, 27). All analytical settings adhered to Brazilian health technology assessment guidelines (21). Cost components included drug acquisition and administration, monitoring, and management of AEs and comorbidities. Costs and benefits were discounted at an annual rate of 5%. A lifetime horizon was selected to adequately capture long-term costs and resource utilization associated with the management of acromegaly as a chronic disease. The cost-effectiveness was assessed using a willingness-to-pay threshold equivalent to three times the gross domestic product (GDP) per capita, estimated in 2022 at R$120,000 (US$22,416), as a reference value to evaluate the value of each alternative (20). In the Brazilian HTA context, however, decision-making in rare diseases may also consider broader factors beyond a single ICER threshold (20). The key model features and settings are summarized in **Table 1**.

**Table 1:** Key model features and settings.

Feature	Description
Population	Adult patients with acromegaly for whom surgery is not an option or has failed to achieve adequate disease control and who are inadequately controlled (IGF-1 > upper limit normal) on treatment with on-label FGSRL (octreotide or lanreotide) regimens.
Intervention	PAS LAR administered at a starting dose of 40 mg by intramuscular injection every 28 days, with potential titration to 60 mg every 28 days according to clinical response, as per the approved prescribing information.
Comparators*	Octreotide 40 mg, lanreotide 120 mg, pegvisomant monotherapy or combination with octreotide 40 mg or lanreotide 120 mg. Pegvisomant is administered at a starting dose of 10 mg daily, with potential titration to 30 mg/day according to clinical response, as per the approved prescribing information.
Perspective	Brazilian Unified Health System (SUS)
Time horizon	Lifetime horizon (40 years) in line with Brazilian HTA guidelines (20)
Discount rate	5% annually for both costs and QALYs, in line with Brazilian HTA guidelines (20)
Outcomes	Total lifetime costs, total LYs, total QALYs, incremental costs, incremental LYs, incremental QALY, ICERs, INMB
Decision tree health states	No control, partial control, full control, death
Markov health states	Full control/partial control,** no control, death
Efficacy source	In the base case, patients transitioned through the model based on IGF-1 normalization. IGF-1 normalization rates were derived from clinical trials and an indirect treatment comparison. In scenario analysis, real-world effectiveness data were tested and a combination of GH <2.5 µg/L and IGF-1 normalization was utilized for comparison with FGSRLs only, as GH is not appropriate for evaluating the efficacy of pegvisomant (10).
Utilities	EQ-5D-based utilities were derived for controlled, partially controlled, and uncontrolled disease, and for subsequent treatment. Health states were defined based on the natural history of the disease. Disutilities were applied to grade 3–4 AEs that occurred in >2% of patients for any treatment. All utility data were retrieved from the literature.
Mortality	The association between acromegaly and its risk of mortality relative to the general population was captured through standardized mortality ratios for each health state, based on the literature.
Costs	Drug acquisition and administration, monitoring, AE management, comorbidities
Sensitivity analyses	Deterministic, probabilistic (1000 iterations), and scenario analysis

*Based on clinical practice, the model assumes a continuous increase in the pegvisomant dose from year 1 until the maximum licensed dose of 30 mg/day is reached. The six-month dose increment was estimated based on ACROSTUDY (47).

**Patients in the partial control and full control health states experience the same treatment pathway and do not transition between these states. For the sake of simplicity in the model description, the combined term full control/partial control is used here.

AE, adverse event; EQ-5D, EuroQol five dimensions; FGSRL, first-generation somatostatin receptor ligand; GH, growth hormone; HTA, health technology assessment; ICER, incremental cost-effectiveness ratio; IGF-1, insulin-like growth factor 1; INMB, incremental net monetary benefit; LY, life year; PAS LAR, pasireotide long-acting release; QALY, quality-adjusted life year.

It is important to note that full control and partial control represent distinct health states within the model and differ with respect to utility values and mortality risk. However, after the initial decision tree phase, patients in these states have the same pathway and do not transition between FC and PC. Thus, the Markov structure combines FC and PC into a single state for pathway modelling, while preserving distinct utility and mortality inputs according to the response category assigned at the end of the decision-tree phase. For the sake of simplicity in the description of the model, the combined term full control/partial control is used in some sections.

## 3 Model health states and transitions

The decision tree phase included three health states, conditioned by biochemical response: no control (NC), partial control, and full control (FC, defined as IGF-1 ≤ 1xULN), and the death state, which represented the background mortality. PC health-state was defined as at least a 50% decrease in IGF-1, according to published criteria for partial biochemical response in acromegaly (28). In the model, however, PC should be interpreted as a technical solution rather than a distinct long-term clinical outcome. It was used to represent patients who were not fully controlled at month 3 but achieved full biochemical control by month 6, under the assumption that they were already deriving measurable health benefits at the 3-month assessment. The Markov phase comprised three possible health states: FC/PC, where patients continued their initial successful therapies; NC, where patients moved after failure of their initial therapies; and death.

All patients entered the initial phase (decision tree) with active acromegaly (IGF-1 above the ULN). **Table 2** presents the baseline characteristics of patients enrolled in the PAOLA clinical trial (29), alongside those of the population used in the model.

**Table 2:** Baseline characteristics of population used in the model.

Characteristic	Weighted average from PAS LAR 40 mg, PAS LAR 60 mg, and FGSRL active control arms (29)
Age	45.84
Proportion of females	56.06%
IGF-1 x ULN	2.77
GH level (µg/L)	13.01
Time since diagnosis of acromegaly (months)	73.92
Previous treatments %	
• Surgery	66.67
• External beam radiation	4.54%
• Gamma-knife therapy	0.5%

FGSRL, first-generation somatostatin receptor ligand; GH, growth hormone; IGF-1, insulin-like growth factor; PAS LAR, pasireotide long-acting release; ULN, upper limit normal.

The model simulated cohorts of a patient pathway for each treatment: PAS LAR and the other comparators. During the decision tree phase, disease control was assessed at three months (first assessment) and six months (second assessment). The six-month assessment corresponds to the efficacy endpoint in PAOLA (19) and is considered sufficient to capture the treatment response to PAS LAR and its comparators. Based on treatment outcomes, patients continued therapy or transitioned to subsequent lines of treatment, as depicted in **Figure 1**. At the first assessment, patients who achieved PC with PAS LAR or PEG regimens were treated with an increased dose of medication to improve their control. All patients classified as achieving PC at month 3 were assumed to transition to full control (FC) by month 6. This follows directly from the model definition of the PC rate, which was calculated as the difference between the proportion of patients achieving FC at 6 months and the proportion achieving FC at 3 months. Patients achieving FC by the second assessment remained on second-line therapy throughout the Markov phase, while those with persistent uncontrolled disease transitioned to subsequent treatment. In the base case it was assumed that there were no PC patients who continued treatment beyond month 6.

The Markov model phase consisted of three health states: FC/PC, NC, or death. The FC/PC state represents a combined pathway state, because patients with full control and partial control continue the same treatment strategy and do not transition between response categories. Patients who entered this phase in FC/PC state continued receiving the treatment until death, with no loss of biochemical control modeled during the Markov phase. Loss of efficacy was set to zero in the base case and was explored only in scenario analysis. It is assumed that achieving a 50% reduction in IGF-1 levels, which defines PC, is sufficient for a patient to remain on treatment. Although this may not represent the desired treatment outcome, it is considered to provide a clinically meaningful health benefit, especially in cases of severe disease and lack of other available treatment options. Patients entered the FC/PC health state with a base dose of PAS LAR (40 mg) or another comparator: LAN (120 mg every 4 weeks), OCT (40 mg every 4 weeks), PEG monotherapy, PEG combined with LAN, or PEG combined with OCT. Patients who entered the Markov phase in the NC state continued receiving subsequent treatment or died. Subsequent lines of treatment included either lifelong medical management or a combination of radiotherapy (RT) and FGSRL. Patients who achieved biochemical control with RT and FGSRL discontinued active treatment, whereas those who failed RT continued FGSRL monotherapy for life. Patients who exited the decision-tree phase due to AEs or lack of disease control received a subsequent treatment until they died (for details see **Supplementary Material 3**). In the scenario analysis that included loss of efficacy, patients who lost biochemical control during the Markov phase were also assumed to move to subsequent treatment. In the base case, the probability of losing control over a patient**’**s lifetime was not included in the model, as no relevant data were identified from published literature.

### 3.1 Model efficacy inputs

Patients transitioned through the model on the basis of biochemical control. In the base case analysis, biochemical control was defined as IGF-1 normalization (IGF-1 ≤ 1xULN) (10), for evaluating PAS LAR against all defined comparators. Based on a consensus statement on therapeutic outcomes in acromegaly (30), partial response was defined as at least a 50% decrease in IGF-1 level. Scenario analysis considered efficacy defined by both IGF-1 normalization and GH <2.5 μg/L (2) for PAS LAR comparison with FGSRLs only, as GH is not appropriate for evaluating the efficacy of PEG.

An SLR was performed to identify studies reporting efficacy and safety inputs for targeted therapies (see **Supplementary Material 1**). IGF-1 remission rates were sourced from three randomized controlled trials (RCTs) that met the SLR’s inclusion criteria (see **Supplementary Material 1**) and allowed for comparative analysis. The PAOLA study (19) reported outcomes for PAS LAR and for FGSRLs, while Trainer et al. (2000) (24) and Trainer et al. (2009) (25) reported outcomes for PEG monotherapy, PEG combination therapy, and placebo.

Direct evidence comparing PAS LAR and FGSRLs was available as both options had been evaluated in one study [PAOLA (19)]. Due to a lack of direct evidence comparing PAS LAR with PEG therapies, only an ITC was possible, employing a connected network of clinical trials (for ITC details see **Supplementary Material 2**). An analysis using a Bucher fixed-effect model (31) was performed using the proportion of patients achieving IGF-1 normalization on their respective treatments, with outcomes presented as odds ratios. The comparative efficacy data resulted from ITC and used in the model are provided in **Supplementary Material 3**.

Due to the heterogeneity of the clinical studies included in the ITC, several assumptions were required. These pertained to the comparability of control arms (serving as a common reference for comparison), the potential influence on treatment efficacy of baseline GH levels and the proportion of patients with prior RT, the assumed equivalence in efficacy between LAN and OCT, and the comparability of treatment efficacy observed between different weeks of therapy. All assumptions are discussed in detail in **Supplementary Material 2**. To address uncertainties associated with the comparative clinical evidence, it was deemed justified to build a scenario where real-world evidence was utilized as an alternative source of efficacy inputs for PAS LAR and PEG regimens in particular. IGF-1 normalization rates for this scenario were extracted from two meta-analyses of real-world evidence in acromegaly (26, 27) that reported the rates for PEG and PAS LAR, respectively, and are provided in **Supplementary Material 3**.

### 3.2 Transition probabilities

The probabilities of achieving FC or PC or remaining in NC during the initial decision three- and six-month assessments are summarized in **Table 3**. Patients with FC or PC continued the current treatment, while patients with NC moved to subsequent treatment. For the transition probabilities, the model applies efficacy data from the PAOLA study, which reports six-month outcomes for PAS LAR 40 mg, PAS LAR 60 mg, and FGSRLs. For PAS LAR, three-month probabilities are derived from PAS LAR 40 mg and six-month probabilities from PAS LAR 60 mg, reflecting dose escalation after three months. PC was not assessed in PAOLA study; therefore, patients achieving FC at six months but not at three months are assumed to have PC at three months. PEG transition probabilities were estimated using an ITC (for details see **Supplementary Material 2**), applying odds ratios versus PAS LAR for three- and six-month outcomes. For the subsequent line involving a combination of RT and medical therapy, 63% of patients achieved disease control 10 years after RT (32).

**Table 3:** Transition probabilities.

Treatment	First assessment (at 3 months)			Second assessment (at 6 months)	
	Achieve FC	Achieve PC	Remain in NC*	PC achieve FC with increased dose	Remain in PC
Base case: IGF-1 ≤ 1xULN. Source: for PEG-based treatments – ITC, remainder – PAOLA study.					
**PAS LAR**	13.18%	12.98%	73.85%	100%	0%
**OCT max. dose**	0.00%	0.00%	100.00%	NA	NA
**LAN max. dose**	0.00%	0.00%	100.00%	NA	NA
**PEG**	6.74%	17.43%	75.83%	100%	0%
**PEG + OCT**	15.66%	12.94%	71.40%	100%	0%
**PEG + LAN**	15.66%	12.94%	71.40%	100%	0%
Scenario analysis: IGF-1 ≤ 1xULN and GH <2.5 μg/L. Source: PAOLA study.					
**PAS LAR**	8.01%	11.99%	80.00%	100%	0%
**OCT max. dose**	0.00%	0.00%	100.00%	NA	NA
**LAN max. dose**	0.00%	0.00%	100.00%	NA	NA
Real-world evidence scenario: IGF-1 only. Source: for PAS LAR – Biagetti et al., 2025 (27), for PEG-based treatments – Leonart et al., 2019 (26).					
**PAS LAR**	35.19%	22.81%	42.00%	100%	0%
**PEG regimens**	37.47%	23.43%	39.10%	100%	0%

FC, full control; GH, growth hormone; IGF-1, insulin-like growth factor 1; LAN, lanreotide; NC, no control; OCT, octreotide; PC, partial control; PAS LAR, pasireotide long-acting release; PEG, pegvisomant; ULN, upper limit of normal. Total life years, total QALYs, and total costs were shown in bold.

### 3.3 Model safety inputs

The model includes grade 3–4 AEs, as these are assumed to cause a greater burden to healthcare providers (in terms of costs) and patients (in terms of quality of life) than grade 1–2 toxicities. AEs were included in the analysis if they occurred in more than 2% of patients. It was assumed that the AEs could occur during the entire time horizon. For each treatment, the incidence of AEs was based on published RCTs. For studies that reported all-grade AEs only (LAN, PEG, and PEG + OCT trials), the incidence of grade 3–4 toxicities was assumed to be proportionally the same as in the PAS LAR study (19). Details on data sources and the incidence of AEs are provided in **Supplementary Material 3**.

### 3.4 Mortality

The increased mortality risk relative to the general population related to acromegaly was modeled using standardized mortality ratios (SMRs) specific to each health state. For fully controlled patients, mortality was assumed to align with that of the general population. Patients with active disease experience elevated mortality risks (6). The SMR for patients remaining in the NC health state was set at 2.40, based on published data (6), while the SMR for the PC health state was estimated as the arithmetic mean of the SMRs for FC and NC, equal to 1.70. An SMR of 2.10 was assumed for subsequent treatment, equal to that reported for acromegaly patients receiving RT (33).

### 3.5 Health-related quality of life

HRQoL in the model was captured through health-state utilities, disutilities associated with treatment-related serious AEs, and comorbidity-related disutilities. Utilities for the FC and NC health states were informed by Liu et al. (2018) (34) in the base case and by Rowles et al. (2005) (35) in scenario analysis. The utility for subsequent treatment was derived from Kyriakakis et al. (2017) (36) and that for PC was assumed to be the arithmetic mean of the FC and NC utilities (**Table 4**).

**Table 4:** Health-state utilities in the model.

Item	Parameter value	Source
Full control base case	0.750	Liu et al., 2018 (34)
Full control scenario	0.810	Rowles et al., 2005 (35)
Partial control base case	0.640	Liu et al., 2018 (34)
Partial control scenario	0.755	Rowles et al., 2005 (35)
No control base case	0.530	Liu et al., 2018 (34)
No control scenario	0.700	Rowles et al., 2005 (35)
Subsequent treatment base case and scenario	0.550	Kyriakakis et al., 2017 (36)

Utility decrements for AEs were derived from published literature (9, 37, 38) and are provided in **Supplementary Material 3**. Individual AE disutilities were multiplied by the AE duration, assumed to be half a month, to calculate the loss of QALYs due to each AE. The QALY losses were further multiplied by the AE probabilities for each comparator (**Supplementary Material 3**) to calculate treatment-specific QALY losses due to AEs.

Although utility data associated with health states derived from the literature account for comorbidities, the model applied additional decrements to capture the different comorbidity profiles of PAS LAR and comparators. This approach was intended to better reflect the progressive nature of the disease and the different comorbidity burden associated with full control, partial control, and no control, rather than assuming that these differences were fully captured by the baseline health-state utility values alone. These utility decrements were derived from published literature (9, 38) and are provided in **Supplementary Material 3**. Comorbidity distributions were determined based on the control criteria and extracted from published studies (7, 39, 40), focusing on cardiovascular (hypertension, arrhythmia, cardiomyopathy), endocrine (diabetes), and respiratory (sleep apnea) conditions. Comorbidity distributions were directly available for the FC and NC states, whereas the distribution for PC was assumed to be the average of FC and NC. The comorbidity distribution for subsequent treatment was calculated as the weighted average of FC and NC values, applying a 63% control rate in subsequent line (32). Comorbidity distributions and utility decrements are provided in **Supplementary Material 3**.

### 3.6 Cost input

All unit costs were expressed in Brazilian reals, while monthly costs were additionally reported in US dollars, based on the exchange rate (41) as of October 10, 2025. In the base case, the model applied costs for drug acquisition and administration, resource use associated with monitoring, AEs, and comorbidities. In scenario analysis, indirect costs associated with productivity losses were also considered.

The prices for each cost category were extracted from official Brazilian tariffs (42) and published literature (43). Values sourced from the SUS Procedures, Medications, Orthoses, Prostheses and from the SIGTAP (Sistema de Gerenciamento da Tabela de Procedimentos, Medicamentos e OPM do SUS – System for Managing the Table of Procedures, Medications, and Special Materials) were adjusted to the real value by applying the correction factor of 2.8, according to recent assessments by CONITEC (Comissão Nacional de Incorporação de Tecnologias no Sistema Único de Saúde – National Committee for Technology Incorporation) (44). All other costs which were not sourced for the current year were inflated to 2025 values using the Consumer Price Index (45) recalculated to the inflation adjustment factor. Cost inputs and sources are provided in **Supplementary Material 3**, section 5.

The model incorporated dosing regimens and drug acquisition costs for PAS LAR and all comparators. While PAS LAR, OCT, and LAN are reimbursed by the Brazilian government (23), PEG is not formally reimbursed; however, its cost may be covered by the government on a case-by-case basis (23). Consequently, PAS LAR, LAN, and OCT are exempt from taxation, whereas the cost of PEG includes an 18% state tax according to the drug price policy in Brazil (46). To enhance the generalizability of the results, a scenario analysis was conducted in which PEG was assumed to be tax-exempt, consistent with other treatments.

Base and increased dosing regimens were applied depending on the treatment strategy and patient response, with costs calculated per unit and aggregated monthly. For PAS LAR, patients in the PC health state received an increased dose of 60 mg to sustain and improve the treatment effect. PEG dosing followed an up-titration approach until the maximum licensed dose of 30 mg/day was reached, with increases estimated from the ACROSTUDY data (47). Combination therapies involving PEG and FGSRLs included the costs of both therapies, adjusted for dosing frequency and duration.

In scenario analysis based on real-world evidence, drug dosing was sourced from data reflecting actual clinical practice. For PAS LAR, dosing information was extracted from publications identified by Biagetti et al. (2025) (27), which reported final doses used in patients receiving PAS LAR as second-line medical therapy. Based on these data, the distribution of patients across specific dose levels (20 mg, 40 mg, and 60 mg administered every 28 days) was established. Patients receiving 20 mg/28 days were reported only sporadically, and no data was available regarding the long-term efficacy of this reduced dose. Consequently, it was assumed that such patients receive the 40 mg/28 days dose in the lifetime perspective. For PEG, dosing was derived from the global ACROSTUDY cohort (48), using a weighted multi-year average of daily doses administered in routine care. The cost per mg of PAS LAR was calculated using weighted average based on the proportion of patients receiving specific dose. For PEG, due to the lack of data on the distribution of patients across different dose level, and the availability of only mean daily dose, the cost per mg was estimated using a simple average of the unit prices of the 10 mg and 15 mg dose strengths.

These inputs are summarized in **Table 5**.

**Table 5:** Dosing and drug acquisition cost inputs.

Health state utilities	Base dose	Unit cost	Monthly cost	Increased dose (applied to PC patients)	Unit cost	Monthly cost
Base case dosing and drug acquisition						
PAS LAR	40 mg/28 days	R$142.50/mg	R$6,196 (US$1,157)	60 mg/28 days	R$117,50/mg	R$7,664 (US$1,432)
OCT	40 mg/28 days	R$114.80/mg	R$4,992 (US$932)	40 mg/28 days	R$114.80/mg	R$4,992 (US$932)
LAN	120 mg/28 days	R$16.10/mg	R$2,100 (US$392)	120 mg/28 days	R$16.10/mg	R$2,100 (US$392)
PEG	13 mg daily	R$28.36/mg	R$11,221 (US$2,096)	20 mg daily	R$28.36/mg	R$17,262 (US$3,225)
PEG + OCT	PEG 15 mg daily	R$28.36mg	R$16,691 (US$3,118)	PEG 20 mg daily	R$28.36/mg	R$21,006 (US$3,924)
	OCT 30 mg/28 days	R$114.80/mg		OCT 30 mg/28 days	R$114.80	
PEG + LAN	PEG 15 mg daily	R$28.36/mg	R$15,047 (US$2,811)	PEG 20 mg daily	R$28.36/mg	R$19,362 (US$3,617)
	LAN 120 mg/28 days	R$16.10/mg		LAN 120 mg/28 days	R$16.10/mg	
Real-world evidence scenario dosing and drag acquisition						
PAS LAR	Average 50.69 mg/28 days	R$129.94/mg	R$6,934 (US$1,295)	No dose escalation in RWE scenario; single dose estimated based on long-term data from published evidence. Average was calculated based on the proportion of patients receiving specific dose.		
PEG	Average 17.33 mg daily	R$28.36/mg	R$14,957 (US$2,794)	No dose escalation in RWE scenario; single dose estimated based on long-term data from published evidence. Average dose was calculated based on ACROSTUDY global data (48).		
PEG + OCT	Average 17.33 mg daily	R$28.36/mg	R$18,701 (US$3,493)	No dose escalation in RWE scenario; single dose estimated based on long-term data from published evidence. Average dose of PEG was calculated based on ACROSTUDY global data (48). OCT dose was assumed the same as in base case.		
	OCT 30 mg/28 days	R$114.80/mg				
PEG + LAN	Average 17.33 mg daily	R$28.36	R$17,057 (US$3,186)	No dose escalation in RWE scenario; single dose estimated based on long-term data from published evidence. Average dose of PEG was calculated based on ACROSTUDY global data (48). LAN dose was assumed the same as in base case.		
	LAN 120 mg/28 days	R$16.10/mg				

IGF-1, insulin-like growth factor 1; LAN, lanreotide; OCT, octreotide; PAS LAR, pasireotide long-acting release; PC, partial control; PEG, pegvisomant; RWE, real-world evidence.

The administration costs in the model are R$0, as there is no cost for oral administration or for intramuscular or subcutaneous injections, according to SIGTAP (42). In line with acromegaly treatment guidelines and recommendations, the model incorporates regular monitoring costs (see **Supplementary Material 3**; **Supplementary Tables 14**, **15**). Unit costs for procedures were sourced from SIGTAP (42) while resource consumption frequencies were informed by clinical recommendations (13, 49, 50). Costs associated with the management of AEs were derived from a nationwide study on healthcare costs in Brazil (43) and validated using SIGTAP data (42) (see **Supplementary Material 3**; **Supplementary Tables 9**, **10**). Comorbidity costs for each health state were sourced from published literature (see **Supplementary Material 3**; **Supplementary Tables 11**–**13**). For subsequent treatment costs (see **Supplementary Material 3**; **Supplementary Table 16**), the model included a one-time cost of RT, and during the first 10 years, based on available local data (DATASUS), the model assumed 29.7% of patients received OCT and 70.3% received LAN. For patients not responding to RT after 10 years, a monthly lifetime cost with FGSRLs was considered.

### 3.7 Sensitivity analysis

The deterministic sensitivity analysis (DSA) examined the impact of varying single inputs to predefined levels on outputs of interest, including incremental QALYs, incremental costs, and INMB. INMB was selected as an incremental result of interest to enable intuitive interpretation of outcomes with PAS LAR being dominant or less effective and less costly versus some comparators. When 95% confidential intervals (CIs) or standard errors (SE) were not available, 10% upward and downward adjustment of inputs was assumed for costs and other inputs (e.g., utility, efficacy). A tornado diagram was generated to visualize the DSA results and indicate which inputs had the largest impact on the outcomes.

In a probabilistic sensitivity analysis (PSA), all uncertain inputs were varied simultaneously by sampling randomly from predefined probability distributions:

- Beta distributions were used to represent uncertainty in efficacy parameters (i.e., transition probabilities at first and second assessment, probability of achieving FC with primary treatment, response loss during initial phase and Markov phase, treatment discontinuation due to AEs, and subsequent-line efficacy proxied by the proportion of patients who achieve biochemical control with RT), likelihood of AEs, comorbidity distribution, utility inputs (related to health states, AEs, and comorbidities). When 95% CIs or SEs were not provided in the input sources, 10% of the point estimate was used to estimate lower and upper range limits.
- Gamma distributions were applied to all costs. SEs for all costs were assumed to be equal to 10% of the point estimate.
- A modified gamma distribution was applied to SMRs to estimate lower and upper range limits.
- Normal distributions represented uncertainty with respect to drug doses, AE duration, and monitoring resource use frequency.
- By default, the PSA included up to 1,000 iterations of the model. A scatterplot of total costs and total benefits was generated for all comparators.

### 3.8 Additional scenario analysis

The following alternative model scenarios were tested:

1. Scenarios testing alternative efficacy assumptions:

- Alternative efficacy source for comparison with PEG regimens (Scenario 1: real-world evidence (27, 48) used instead of ITC results).
- PAS LAR efficacy source based on C2305 study (29) (Scenario 2). A study outside the PICO (population, interventions, comparators, outcomes) criteria was included, presenting first-line efficacy for PAS LAR, as no alternative source for PAS LAR efficacy meeting the PICO criteria was identified.
- PAS LAR efficacy source for comparison with FGSRLs based on C2413 (51) study with biochemical control criteria GH <1 μg/l and normalized IGF-1 (Scenario 3). C2413 is a phase IIIb open-label trial that studied adult patients with acromegaly who did not achieve disease control on maximally approved doses of FGSLRs (LAN, OCT) and switched to PAS LAR 40 mg/28 days.
- Alternative biochemical control criteria for comparison with FGSRLs (Scenario 4: GH <1 µg/L and normalized IGF-1).
- Partial control patients continue treatment beyond month 6 (Scenario 5).
- Treatment effect loss assumed from year 1 (Scenario 6).

2. Scenarios testing the PEG dose escalation assumption:

- Fixed PEG dose over lifetime (without up-titration to the maximum 30 mg/day; scenario 7).
- Ceiling PEG dose based on RWE data (maximum PEG dose based on RWE data, scenario 8).
- Addition of FGSRLs to PEG monotherapy (47) (proportion of patients receiving add-on FGSRLs instead of dose increase, Scenario 9).

3. Scenarios varying PEG costs:

- PEG price without VAT (scenario 10).
- PEG lower-bound price (52) (PEG price corridor, scenario 11).
- PEG upper-bound price (52) (PEG price corridor, scenario 12).
- PEG hypothetical reimbursed price (PEG price assuming routine reimbursement based on the reimbursement submission, scenario 13) (53).

4. Scenarios related to clinical assumptions:

- Chronic PAS LAR-related hyperglycemia management (54) (long-term antihyperglycemic treatment for PAS LAR-induced hyperglycemia, scenario 14).
- Delayed RT effect and adjusted RT outcome (Scenario 15: 20 years).

5. Scenarios testing utilities assumptions:

- Alternative source (35) for health state utility values (Scenario 16).
- Exclusion of disutilities from AEs (Scenario 17).
- Exclusion of disutilities from comorbidities (Scenario 18).
- Partial control comorbidities closer to full control comorbidities (Scenario 19).

6. Scenarios exploring AE assumptions:

- Proportion of PEG’s AEs being severe set as 50% of PAS LAR proportion (scenario 20).
- Proportion of PEG’s AEs being severe set as 200% of PAS LAR proportion (scenario 21).

7. Scenarios on general modelling assumptions:

- Shorter model time horizons (Scenario 22: 20 years; Scenario 23: five years).
- Consideration of a societal perspective that accounted for productivity losses attributable to acromegaly (Scenario 24).
- Discount rates of 0%, 1.5%, and 10% (Scenarios 25–27).

## 4 Results

### 4.1 Base case

All results are reported in Brazilian reals and US dollars, based on the exchange rate (41) as of October 10, 2025 and are presented in **Table 6**.

**Table 6:** Base case results.

Outcome	PAS LAR	OCT	LAN	PEG only	PEG + OCT	PEG + LAN
Health outcomes						
**Total LYs**	**14.4875**	**14.1117**	**14.1117**	**14.4381**	**14.5005**	**14.4586**
**Full control**	3.7838	0.0000	0.0000	3.2786	3.9165	3.4952
**Partial control**	0.0312	0.0000	0.0000	0.0406	0.0302	0.0285
**No control**	0.2500	0.2500	0.2500	0.2500	0.2500	0.2500
**Subsequent treatment**	10.4226	13.8617	13.8617	10.8690	10.3037	10.6849
**Total QALYs**	**6.0289**	**4.8117**	**4.8116**	**5.8606**	**6.0646**	**5.9297**
**Full control**	2.3743	0.0000	0.0000	2.0500	2.4516	2.1879
**Partial control**	0.0152	0.0000	0.0000	0.0197	0.0147	0.0139
**No control**	0.0873	0.0874	0.0874	0.0868	0.0869	0.0869
**Subsequent treatment**	3.5520	4.7243	4.7243	3.7040	3.5114	3.6410
Costs						
**Total costs**	**R$774,946 (US$144,760)**	**R$578,476 (US$108,059)**	**R$569,800 (US$106,439)**	**R$1,313,891 (US$245,435)**	**R$1,754,570 (US$327,754)**	**R$1,557,489 (US$290,939)**
**Drug acquisition**	R$716,711 (US$133,882)	R$522,563 (US$97,615)	R$513,887 (US$95,994)	R$1,249,955 (US$233,492)	R$1,691,110 (US$315,899)	R$1,494,815 (US$279,231)
**Administration**	R$0 (US$0)	R$0 (US$0)	R$0 (US$0)	R$0 (US$0)	R$0 (US$0)	R$0 (US$0)
**Monitoring**	R$19,999 (US$3,736)	R$17,263 (US$3,225)	R$17,263 (US$3,225)	R$25,202 (US$4,708)	R$24,739 (US$4,621)	R$23,956 (US$4,475)
**Comorbidity**	R$38,163 (US$7,129)	R$38,616 (US$7,213)	R$38,616 (US$7,213)	R$38,227 (US$7,141)	R$38,146 (US$7,126)	R$38,197 (US$7,135)
**AE management**	R$73 (US$14)	R$34 (US$6)	R$35 (US$6)	R$506 (US$95)	R$576 (US$108)	R$521 (US$97)
Incremental results						
**Incremental LYs**	NA (reference treatment)	0.3757	0.3757	0.0494	-0.0130	0.0289
**Incremental QALYs**		1.2172	1.2172	0.1683	-0.0358	0.0992
**Incremental costs**		R$196,470 (US$36,701)	R$205,146 (US$38,321)	-R$538,945 (-US$100,675)	-R$979,624 (-US$182 994)	-R$782,542 (-US$146,179)
**ICER per LY**		R$522,886 (US$97,675)	R$545,975 (US$101,988)	PAS is dominant	PAS is less costly, less effective	PAS is dominant
**ICER per QALY**		R$161,411 (US$30,152)	R$168,538 (US$31,483)	PAS is dominant	PAS is less costly, less effective	PAS is dominant
**INMB**		-R$50,406 (-US$9,416)	-R$59,081 (-US$11,036)	R$559,138 (US$104,447)	R$975,333 (US$182,192)	R$794,443 (US$148,402)

AE, adverse event; ICER, incremental cost-effectiveness ratio; INMB, incremental net monetary benefit; LAN, lanreotide; LY, life year; NA, not applicable; OCT, octreotide; PAS LAR, pasireotide long-acting release; PEG, pegvisomant; QALY, quality-adjusted life year. Total life years, total QALYs, and total costs were shown in bold.

PAS LAR compared to PEG monotherapy resulted in similar total life years (LYs) (14.4875 vs 14.4381) and total QALYs (6.0289 vs 5.8606), with patients spending more time in the FC state (3.7838 vs 3.2786 years). The total costs of PAS LAR therapy were substantially lower [R$774,946 (US$144,760) vs R$1,313,891 (US$245,435)], primary due to differences in drug acquisition costs. As a result, PAS LAR was dominant over PEG monotherapy, offering improved health outcomes at a lower cost.

PAS LAR compared to PEG in combination with OCT resulted in comparable total LYs (14.4875 vs 14.5005) and QALYs (6.0289 vs 6.0646), with patients spending a comparable amount of time in the FC state (3.7838 vs 3.9165 years). The total cost of PAS LAR remained over two times lower [R$774,946 (US$144,760) vs R$1,754,570 (US$327,754)], making it a more cost-effective option compared to the PEG and OCT combination. When compared to the combination of PEG with LAN, PAS LAR achieved slightly higher QALYs (6.0289 vs 5.9297) and comparable LYs (14.4875 vs 14.4586), with patients spending more time in the FC state (3.7838 vs 3.4952 years). The total cost of PAS LAR therapy was substantially lower [R$774,946 (US$144,760) vs R$1,557,489 (US$290,939)] and as a result, PAS LAR was dominant over the PEG and LAN combination, offering similar health outcomes at a significantly lower cost.

PAS LAR compared to FGSRLs improved overall LYs and QALYs by achieving better disease control. Patients treated with PAS LAR spent more time in the FC state (3.78 years vs 0.00 years for FGSRLs) and had higher total QALYs (6.0289 vs 4.8116 for LAN and 4.8117 for OCT). The total costs of PAS LAR (R$774,946 [US$144,760]) were higher than those of OCT (R$578,476 [US$108,059]) and LAN (R$569,800 [US$106,439]), primarily driven by drug acquisition costs. The resulting ICERs were R$161,411 (US$30,152) per QALY versus OCT and R$168,538 (US$31,483) per QALY versus LAN. Their interpretation should be made in the context of rare-disease decision-making in Brazil, where considerations beyond the reference threshold may also be relevant.

**Figure 2:**
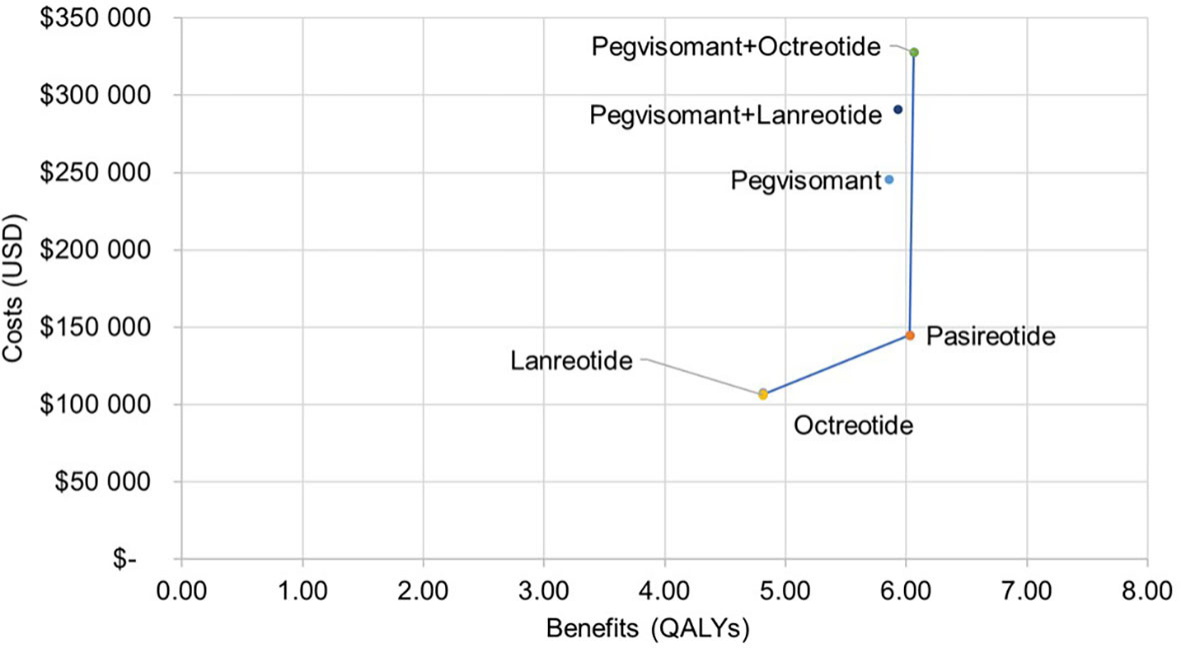
Cost-effectiveness frontier.

The cost-effectiveness frontier was formed by LAN, PAS LAR, and PEG + OCT. All evaluated treatment strategies were plotted according to their total costs and QALYs (**Figure 2**).

PAS LAR is positioned in the lower-right area of the plot, indicating a favorable combination of high effectiveness and low cost. PEG regimens, particularly PEG in combination with FGSLRs, appear in the upper-right quadrant, reflecting higher effectiveness but substantially increased costs. The frontier connects non-dominated strategies.

### 4.2 Deterministic sensitivity analysis results

When PAS LAR is compared with PEG monotherapy, the most influential parameters included the probability of treatment discontinuation due to AEs, transition probabilities from NC to partial or FC, and assumptions regarding PEG dosing. Finally, when PAS LAR is compared with combined therapy of PEG with FGSRLs, DSA identified the most influential parameters to be the probability of treatment discontinuation due to AEs, transition probabilities from NC to partial or FC, and assumptions regarding the base doses of drugs.

In the comparison of FGSRLs and PAS LAR, DSA identified the utilities for the FC health state and subsequent treatment, as well as the dosing of PAS LAR, as the most influential parameters affecting model outcomes.

**Figure 3:**
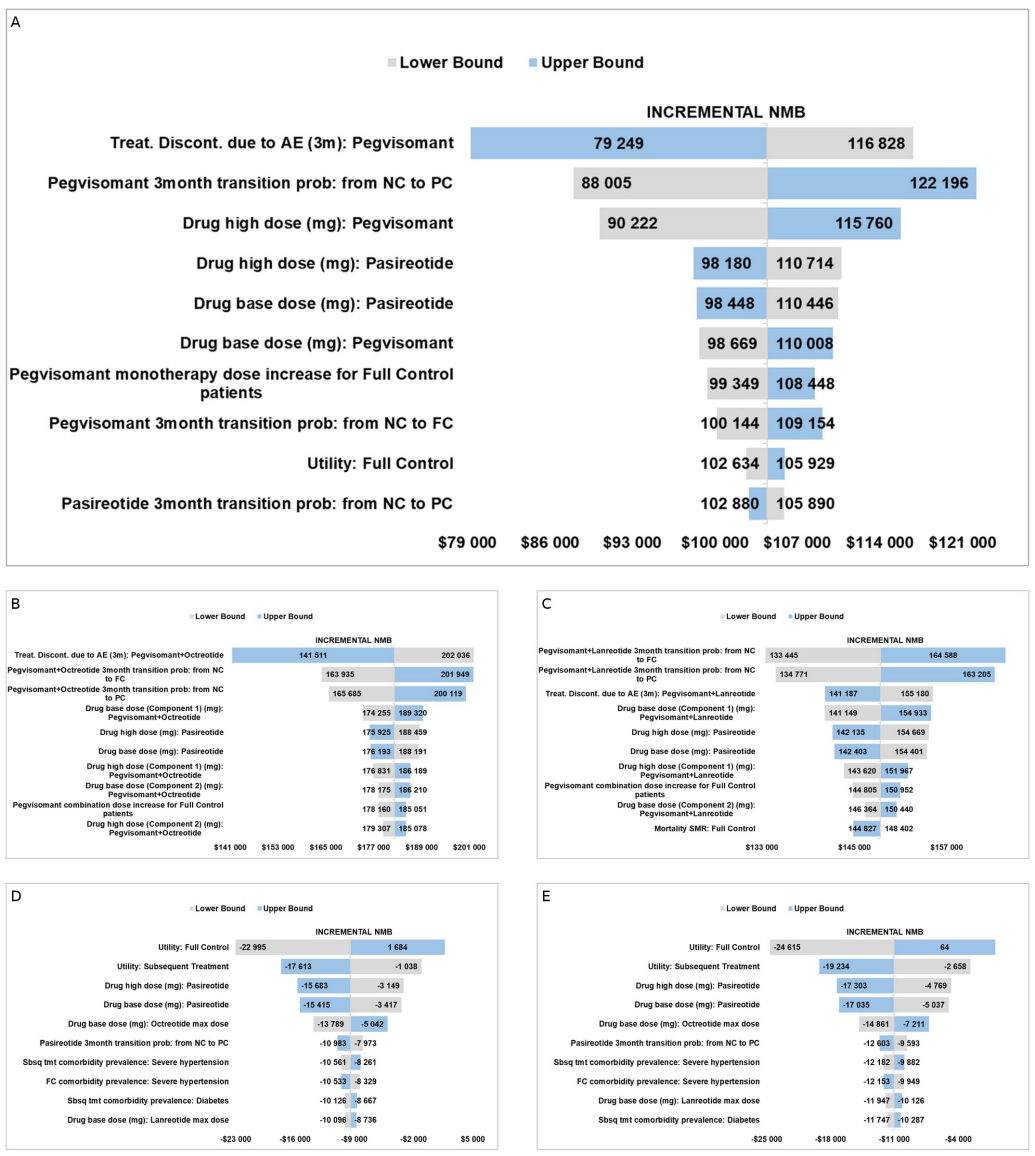
DSA results for INMB of PAS LAR vs **(A)** PEG, **(B)** PEG + OCT, **(C)** PEG + LAN, **(D)** OCT, **(E)** LAN.

Tornado diagrams presenting the parameters with the greatest impact on INMB across all treatment strategies are provided in **Figure 3**. Diagrams presenting the parameters with the greatest impact on QALYs and total costs are provided in **Supplementary Material 4**; **Supplementary Figures 1**–**5**.

### 4.3 Probabilistic sensitivity analysis results

**Figure 4:**
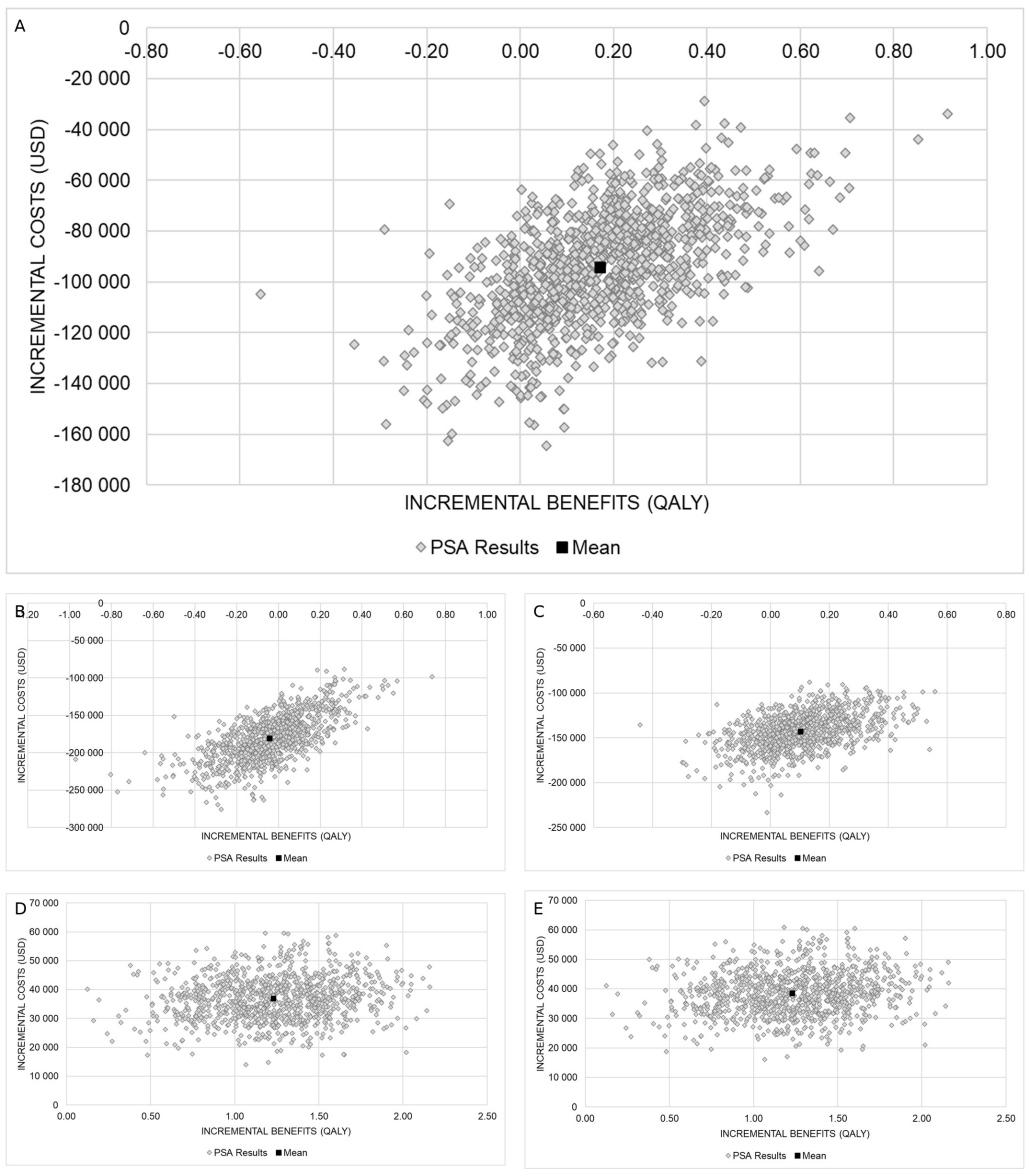
PSA cost-effectiveness scatterplots for PAS LAR vs **(A)** PEG, **(B)** PEG + OCT, **(C)** PEG + LAN, **(D)** OCT, **(E)** LAN.

In all PSA simulations, PAS LAR exhibited a 100% probability of being cost-effective versus PEG regimens across willingness-to-pay (WTP) thresholds ranging from US$0 to US$100,000. PAS LAR was consistently less costly than PEG regimens; however, the incremental clinical benefit varied, favoring either PAS LAR or PEG. Compared with OCT and LAN, PAS LAR surpassed a 50% probability of being cost-effective at a WTP threshold of approximately R$165,953 (US$31,000), above the Brazilian reference threshold of R$120,000 (US$22,416)/QALY. PAS LAR consistently demonstrated higher effectiveness, although it was associated with increased costs. Cost-effectiveness scatterplots are presented in **Figure 4** and cost-effectiveness acceptability curves are provided in **Supplementary Material 5**; **Supplementary Figures 1**–**5**.

### 4.4 Scenario analysis

Implementing alternative efficacy assumptions where real-world effectiveness was considered for PAS LAR and PEG regimens (real-world evidence, scenario 1), show that PAS LAR is dominant over PEG regimens, with incremental costs of -R$1,588,165 (-US$296,669) and a QALY gain of 0.1064. Detailed results for this key scenario are presented in tabular form (**Table 7**).

**Table 7:** RWE scenario results.

Outcome	PAS LAR	PEG regimens
Total LYs	14.9455	14.9176
Total QALYs	7.5134	7.4070
Total costs	R$1,013,163 (US$189,259)	R$1,952,050 (US$364,643)
Incremental LYs	Reference	0.0279
Incremental QALYs		0.1064
Incremental costs		-R$938,887 (-US$175,384)
ICER (per LY)		PAS is dominant
ICER (per QALY)		PAS is dominant

ICER, incremental cost-effectiveness ratio; LY, life year; PAS LAR, pasireotide long-acting release; PEG, pegvisomant; QALY, quality-adjusted life year.

The remaining scenarios related to alternative efficacy assumptions showed moderate sensitivity of the results to these inputs. In Scenario 2, where PAS LAR efficacy was based on the C2305 study, PAS LAR remained dominant versus PEG and PEG + LAN, while remaining less costly but less effective versus PEG + OCT. When PAS LAR efficacy versus FGSRLs was informed by the C2413 study using the GH <1 μg/L and normalized IGF-1 criterion (Scenario 3), incremental QALYs were markedly reduced, while ICERs remained in a similar range. Applying the alternative biochemical control definition for FGSRL comparison only (Scenario 4) yielded ICERs of R$167,099/QALY and R$176,363/QALY versus OCT and LAN, respectively. Allowing partial control patients to remain on treatment beyond month 6 (Scenario 5) increased incremental QALYs and costs across PEG-based comparators; PAS LAR remained dominant versus PEG and PEG + LAN and less costly but less effective versus PEG + OCT. In the FGSRL comparisons, this scenario led to ICERs increasing to R$208,890/QALY versus OCT and R$214,336/QALY versus LAN. Assuming treatment effect loss from year 1 (Scenario 6) improved the PAS LAR relative results versus PEG-based regimens, with PAS LAR becoming dominant also versus PEG + OCT.

The scenarios testing the PEG dose escalation showed that the results were sensitive to this assumption. Compared with the base case, fixing the PEG dose over lifetime (Scenario 7) reduced the cost difference between PAS LAR and PEG regimens, with incremental costs changing from -R$538,945 to -R$338,229 versus PEG, from -R$979,624 to -R$597,288 versus PEG + OCT, and from -R$782,542 to -R$441,528 versus PEG + LAN, while PAS LAR remained dominant versus PEG and PEG + LAN and less costly but less effective versus PEG + OCT. Applying a ceiling PEG dose based on RWE data (Scenario 8) also reduced the cost difference, with incremental costs of -R$369,083 versus PEG, -R$643,984 versus PEG + OCT, and -R$483,178 versus PEG + LAN. Similarly, adding FGSRLs to a proportion of patients on PEG monotherapy with a ceiling PEG dose based on RWE (Scenario 9) resulted in incremental costs of -R$453,277 versus PEG, -R$643,984 versus PEG + OCT, and -R$483,178 versus PEG + LAN. ICERs.

As expected, alternative PEG cost assumptions primarily affected the results by modifying the total costs of PEG-based treatment strategies. Applying the PEG lower-bound price (Scenario 11) reduced the cost difference between PAS LAR and PEG regimens, with incremental costs of -R$417,151 versus PEG, -R$877,220 versus PEG + OCT, and -R$691,088 versus PEG + LAN. By contrast, using the PEG upper-bound price (Scenario 12) increased the cost difference in favor of PAS LAR, with incremental costs of -R$591,632 versus PEG, -R$1,050,327 versus PEG + OCT, and -R$845,887 versus PEG + LAN. The hypothetical reimbursed PEG price (Scenario 13) produced the smallest cost difference versus PAS LAR among the PEG cost scenarios, with incremental costs of -R$46,281 versus PEG, -R$332,513 versus PEG + OCT, and -R$202,594 versus PEG + LAN, although PAS LAR remained cost saving in all PEG-based comparisons. Compared with the base case, removing VAT from PEG prices (Scenario 10) resulted in incremental costs changing from -R$538,945 to -R$386,025 versus PEG, from -R$979,624 to -R$782,811 versus PEG + OCT, and from -R$782,542 to -R$601,294 versus PEG + LAN.

The scenarios related to clinical assumptions had a limited overall impact on the model outcomes, although the direction and magnitude of change differed between the two scenarios. Compared with the base case, inclusion of chronic PAS LAR-related hyperglycemia management (Scenario 14) slightly worsened cost-effectiveness results of PAS LAR by increasing its treatment-related costs. In PEG-based comparisons, this reduced the cost advantage of PAS LAR, with incremental costs changing from -R$538,945 to -R$536,928 versus PEG, from -R$979,624 to -R$977,607 versus PEG + OCT, and from -R$782,542 to -R$780,526 versus PEG + LAN, while PAS LAR remained dominant versus PEG and PEG + LAN and less costly but less effective versus PEG + OCT. In comparisons versus FGSRLs, the same scenario slightly increased the ICERs from R$161,411/QALY to R$163,068/QALY versus OCT and from R$168,538/QALY to R$170,194/QALY versus LAN, indicating only a marginal deterioration in cost-effectiveness relative to the base case. By contrast, delayed RT effect and adjusted RT outcomes (Scenario 15) led to more favorable results for PAS LAR overall, improving its cost-effectiveness profile versus both PEG-based regimens and FGSRLs.

The remaining utility and disutility scenarios showed that the model results were only minimally sensitive to these assumptions. Excluding disutilities for comorbidities (Scenario 18) led to only minor differences in incremental QALYs, with no meaningful impact on costs or overall conclusions. Similarly, assuming that comorbidity burden in the partial control state was closer to that in the full control state (Scenario 19) produced results that were virtually identical to the base case. The scenarios exploring AE assumptions (Scenarios 20 and 21) showed that varying the proportion of severe PEG-related adverse events had a negligible impact on model outcomes. Reducing the severe AE rate for PEG (Scenario 20) slightly worsened the QALY results for PAS LAR versus PEG-based regimens, whereas increasing it (Scenario 21) slightly improved them; however, neither scenario had a meaningful impact on costs or cost-effectiveness conclusions. The scenarios on general modelling assumptions (Scenarios 22–27) showed the expected pattern. Shortening the model time horizon to 20 years (Scenario 22) resulted in lower incremental QALYs and costs across all comparators but maintained similar cost-effectiveness conclusions. A 5-year horizon (Scenario 23) led to substantially lower QALYs and costs and more favorable ICERs versus FGSRLs, although PAS LAR remained dominant in comparisons with PEG and PEG + LAN and less costly versus PEG + OCT. Scenario 24 (societal perspective) indicated that changing the perspective had a minimal impact on model outcomes. Scenarios varying the discount rate (0%, 1.5%, and 10%; Scenarios 25–27) showed a clear pattern: lower discount rates led to higher incremental QALYs and costs, while a higher discount rate resulted in lower QALYs and costs. Despite these shifts, PAS LAR consistently remained dominant or cost saving in PEG-based comparisons, while the conclusions versus FGSRLs were not materially altered. The results of all remaining scenario analyses, including detailed outcomes for each modeled case, are provided in **Supplementary Material 4**.

## 5 Discussion

This study demonstrates the cost-effectiveness of PAS LAR as a second-line medical therapy for adult acromegaly patients who are not controlled with FGSRLs in Brazil. A hybrid decision-tree and Markov model was developed to reflect clinical practice, incorporating time intervals related to the assessment of treatment response and continuation and dose titration across therapies. As all relevant cost categories have been considered and resource use was differentiated according to disease control, the resulting estimates of respective treatment costs are comprehensive and considered to adequately reflect clinical practice.

Several strengths of this analysis can be highlighted. First, it provides a comprehensive evaluation of all second-line pharmacological options available at the time of the analysis for the treatment of adult acromegaly patients who are not controlled with FGSRLs in Brazil. Second, this study is the first economic study to employ relative clinical efficacy estimated for PAS LAR and PEG treatments, adjusted for prognostic factors through indirect comparison using control arms from distinct RCTs. It is also the first analysis to apply IGF-1 normalization rates for PAS LAR and PEG derived from real-world evidence (26, 27). In addition, the model can accommodate both definitions of biochemical criteria (IGF-1 only; GH and IGF-1) used in treatment guidelines and clinical trials (26, 27).

The most recent published cost-effectiveness models applied Markov structures that incorporated three health states: controlled, uncontrolled, and death. Two models, Brue et al. (2021) (also described in Raverot et al., 2025) (55, 56) and Peral et al. (2020) (57), evaluated the cost-effectiveness of second-line pharmacological treatments from French and Spanish payer perspectives, respectively. Leonart et al. (2021) (58) analyzed the cost-effectiveness of medical treatment strategies, comprising first- and second-line therapies, from a Brazilian perspective. Like our analysis, all three studies assumed that if a controlled state is achieved by the end of the last response evaluation, patients remain on treatment until death. All models covered lifetime progression of the disease. None of the studies assumed treatment discontinuation due to loss of efficacy. Treatment discontinuation due to AEs was modeled only in Leonart et al. (2021) (58). All the cost-effectiveness studies showed PAS LAR and PEG treatments to be more expensive than FGSRLs but also to provide additional health benefits. Similarly to our study, Leonart et al. (2021) (58) concluded that the cost-effectiveness of PAS LAR and PEG regimens was comparable in Brazil. On the other hand, Brue et al. (2021) (55) and Peral et al. (2020) (57) found PEG regimens to be more cost-effective than PAS LAR, with PEG generating more QALYs.

One significant difference between the cost-effectiveness studies was related to the impact of non-pharmacological costs on the results, particularly hyperglycemia and diabetes management costs, as well as treatment of comorbidities. In our study, even though a large proportion of PAS LAR patients required antidiabetic treatment, total non-pharmacological costs did not differ much between PAS LAR and PEG. A similar approach was reported by Leonart et al. (2021) (58). Conversely, in Brue et al. (2021) (55) and Peral et al. (2020) (57), these differences were substantial and ultimately altered the perspective on total therapy costs. The study by Venegas-Moreno et al. (2025) (59) also confirmed that, in clinical practice, deterioration in blood glucose control with PAS LAR was generally mild and well controlled with anti-diabetics drugs and did not generate a significant cost burden for payers, since acromegaly patients were already monitored by endocrinology specialists, who could coordinate multimodal care for type-2 diabetes.

Other major differences in the cost-effectiveness conclusions are likely due to key model efficacy inputs. They are not specific to the Brazilian healthcare context. According to Brue et al. (2021) (55) and Peral et al. (2020) (57), PEG regimens offered markedly greater disease control than PAS LAR in contrast to Leonart et al. (2021) (58) and to our study, which showed similar disease control for PEG regimens and PAS LAR, based on relative effectiveness results derived from an ITC. A similar level of disease control for PAS LAR and PEG was validated by scenario analysis incorporating the effectiveness derived from real-world evidence (26, 27). Importantly, the transition probabilities used in the model for PEG regimens were derived from the ITC and were not taken directly as crude response rates from the trials. These probabilities were estimated by applying the relative treatment effects from the ITC to the PAS LAR response profile observed in the PAOLA study. As a result, because the PAOLA study had more severe population and showed lower response rates than the PEG trials, the estimated PEG probabilities of achieving IGF-1 control in the model were also lower than the crude response proportions reported by Trainer et al. (2000) (24) and Trainer et al. (2009) (25).

It is worth noting that real-world studies (48, 60), including the multicenter, multinational ACROSTUDY, reported IGF-1 control rates of approximately 53–55% with PEG regimens. These rates were markedly lower than those observed in clinical studies (24, 25). In contrast, real-world studies of PAS LAR (61–63) consistently reported higher IGF-1 normalization rates than in clinical studies (19, 29). In the case of PEG, this might be explained to some extent by compliance issues reported in clinical practice (48). It is also likely that patients selected for the PAS LAR clinical studies had a greater disease burden and a less favorable prognosis than those included in the PEG studies (19, 24, 25). The analysis of patients’ baseline characteristics from the clinical studies shows large heterogeneity, including in baseline GH levels and the proportions of patients with previous RT, which are commonly reported prognostic factors of biochemical control (39, 64, 65). Notably, in the PAOLA study the baseline GH levels were 17.6 µg/L and 12.1 µg/L in the PAS LAR 40 mg and PAS LAR 60 mg arms, respectively. In contrast, GH levels in the PEG arms varied between 3.0 µg/L and 4.2 µg/L in Trainer et al. (2009) (25) and between 7.8 µg/L and 11.5 µg/L in Trainer et al. (2000) (24), indicating that patients might have had less severe acromegaly than those in the PAOLA study. Only 3–5% of PAS LAR patients had previously received RT, compared to 42–66% in Trainer et al. (2000) (24). For this reason, it was not considered appropriate to extract IGF-1 values from the studies of PAS LAR and PEG and apply them in the present cost-effectiveness model. Instead, baseline differences in patient characteristics across the three trials were assumed to be mitigated by the ITC employing relative effectiveness established via common comparators (31, 66).

Due to the uncertainties related to the relative efficacy outcomes from our ITC, the model results were validated with real-world evidence for PAS LAR and PEG. Leonart et al. (2019) (26) and Biagetti et al. (2025) (27) report on SLRs and meta-analyses for PEG and PAS LAR, respectively, in terms of IGF-1 control rates in acromegaly patients in real-world clinical practice. The relative efficacy results for PAS LAR and PEG regimens derived from our ITC are consistent with these published studies, which validate crucial clinical model inputs. Both the ITC and real-world evidence show comparable rates of IGF-1 normalization for PAS LAR and PEG.

Despite its strengths, the study presents some limitations that should be considered. First, the model simplifies clinically relevant long-term treatment pathways, including persistence of partial control, loss of biochemical control over time, long-term PEG dose evolution or FGSRLs additions. In addition, the model does not simulate subsequent treatment pathways separately due to data gaps. Instead, it assumes a generalized “basket” of subsequent treatments, comprising medical therapy and RT. These assumptions reflect the constraints of available data; however, they may oversimplify the heterogeneity of outcomes associated with various treatments. Second, all endpoints related to treatment objectives as described in clinical guidelines (10) were not considered as biochemical control was the sole determinant for treatment continuation. In particular, tumor size reduction, headaches, HRQoL, and LY benefits were not incorporated. Third, PAS LAR-related hyperglycemia was captured in the base case mainly as an adverse event rather than as a treatment-specific long-term metabolic condition, which may underestimate persistent management costs in some patients. In addition, the present analysis was conducted from the perspective of the Brazilian Unified Health System (SUS) and reflects local drug prices, reimbursement and procurement mechanisms, and treatment pathways. Therefore, these results are not directly transferable to other healthcare systems without recalibration to local conditions.

Several scenario analyses were specifically performed to test strong assumptions of the model. The scenario allowing partial control to persist beyond month 6 addressed the clinically important concern that not all patients with partial biochemical improvement will subsequently achieve full control yet may continue the current medical treatment. This scenario changed the quantitative results, but it did not alter the overall model conclusions. Likewise, the escape scenario examined the assumption of sustained long-term control and indicated that the no-escape base-case assumption was not a major driver of the model conclusions. Another source of uncertainty concerned PEG regimens, both in terms of acquisition cost and long-term dosing. From the Brazilian SUS perspective, PEG cost is difficult to define because the drug is not routinely reimbursed and may be accessed through heterogeneous pathways. For this reason, alternative price scenarios were explored to distinguish current Brazilian access conditions from a hypothetical routinely reimbursed setting. These analyses confirmed that PEG pricing is an important driver of the magnitude of cost differences in PEG-based comparisons, although it did not overturn the main conclusions. Similarly, the base-case assumption of progressive PEG dose escalation represented a pragmatic simplification intended to reflect possible long-term treatment intensification. This uncertainty was also tested in scenario analyses, which showed that the PEG-related results were sensitive to dose assumptions, again mainly quantitatively rather than qualitatively. Finally, PAS LAR-related hyperglycemia was captured in the base case primarily as an adverse event rather than as a treatment-specific chronic metabolic condition. This approach was consistent with available evidence suggesting that hyperglycemia associated with PAS LAR is often manageable in clinical practice (54, 67), but it could still underestimate persistent treatment-related burden in some patients. A dedicated scenario therefore explored long-term antihyperglycemic treatment, and its limited impact on outcomes suggests that this assumption is unlikely to materially affect the conclusions of the model.

While acknowledging this limitation, it should be noted that cost-effectiveness models are simplified representations of a disease course and focus on selected disease aspects to allow comparability of all medical interventions. The deterministic and probabilistic sensitivity analyses, however, confirm the robustness of the results, which consistently demonstrate the clinical benefits and economic value of PAS LAR. Nevertheless, the outcomes seem to be sensitive to variations in utilities and to dose-related costs. In addition, although the ICERs for PAS LAR versus FGSRLs were above the reference threshold of R$120,000/QALY, their interpretation should consider the broader HTA context for rare diseases in Brazil. In this setting, factors such as disease severity, unmet need, quality-of-life impact, uncertainty, and equity may also be relevant alongside the ICER. Indeed, despite ICERs exceeding this reference threshold, the Brazilian Ministry of Health has already incorporated pasireotide into the SUS for the treatment of acromegaly in patients who do not respond adequately to FGSRL such as octreotide or lanreotide. In this context, we consider PAS LAR to remain a relevant and potentially cost−effective option, even though the ICER surpasses the base reference threshold.

Future research should aim to address data gaps related to comparative efficacy in real-world clinical practice, the use of subsequent treatments, and the refinement of comorbidity and utility data to enable robust comparisons of acromegaly therapies.

## 6 Conclusions

The findings of the presented cost-effectiveness analysis suggest that PAS LAR is cost-saving compared to PEG-based strategies. PAS LAR was dominant versus PEG monotherapy and PEG + LAN, generating slightly higher QALYs at lower costs, whereas versus PEG + OCT it was less costly and associated with a marginal QALY decrement. While PEG + OCT therapy yield slightly higher QALYs, the substantial increase in drug acquisition costs results in less favorable cost-effectiveness profiles relative to PAS LAR.

In Brazil, PAS LAR is currently reimbursed only for a restricted subgroup of acromegaly patients who present with tumor remnants. However, our study demonstrates that the treatment improves both life expectancy and quality of life in the modeled population of all adult patients inadequately controlled on FGSRLs. Given the substantial disease burden associated with acromegaly, as reflected by an elevated SMR and a high prevalence of comorbidities, these findings highlight the critical need to address the unmet medical challenges faced by patients with persistent, uncontrolled disease. Consequently, they may provide valuable evidence to support future considerations of broader reimbursement for PAS LAR in Brazil and in other countries facing similar reimbursement conditions.

## Data Availability

The original contributions presented in the study are included in the article/**Supplementary Material**. Further inquiries can be directed to the corresponding author.
