# Supplementary material for: Cost−effectiveness of pasireotide as second−line therapy for adult acromegaly patients from the perspective of the Brazilian Unified Health System
Source: Front Endocrinol (Lausanne). 2026 Aug 5;17:1771580. doi: 10.3389/fendo.2026.1771580 (PMC13487751; doi:10.3389/fendo.2026.1771580)
Supplement: Supplementary file 1 [file SupplementaryFile1.docx]

# Systematic literature review details

Systematic literature review aimed to identify clinical trials of treatments for adults with acromegaly and was conducted in accordance with the quality standards outlined in established guidelines [1,2], including transparent documentation of the review methodology, search strategy, study attrition and results. A systematic literature search on papers published between January 2013 and 2021 covered the following databases: MEDLINE, MEDLINE In-Process, MEDLINE® Daily and other non-indexed citations, Embase, The Cochrane Library (CENTRAL and CDSR). In addition, conference abstract databases were searched, including ESE Conference, Endocrine Society Conference and ENEA Conference. Details of all databases searched, along with the date limits applied, are provided in Table 1 below.

**Table 1. Database and Conference Sources search in SLR.**

| **Electronic Databases (via Ovid.com)** | **Search Dates** |
| --- | --- |
| - MEDLINE, MEDLINE In-Process, MEDLINE® Daily and other non-indexed citations (via Ovid) - Embase (via Ovid) - The Cochrane Library (CENTRAL and CDSR, via Ovid) | - May 2013 - August 2014 - 1 January 2014 to 10 February 2021 |
| - ESE Conference (https://www.ese-hormones.org/) - Endocrine Society Conference (https://www.endocrine.org/) - ENEA Conference (https://eneassoc.org/) | - Past two years (2019, 2020) |
| - Reference checking of previously published SLRs and meta-analyses (2017 onwards) | - Not applicable |
| Literature searches were conducted in electronic databases, conference proceedings and other sources to identify clinical trials of treatments for adults with acromegaly  Abbreviations: ESE, European Society of Endocrinology; ENEA, European NeuroEndocrine Association; SLR, systematic literature review | |

Studies were selected for inclusion in the SLR based on the populations, interventions and comparators, outcomes, and study design (PICOS) framework. The targeted population consisted of adult patients with acromegaly for whom surgery was not an option or had not been curative, and the subpopulation of interest was patients, who were also not adequately controlled on first line medical treatments. Outcomes of interest were related to the efficacy, safety, and health-related quality of life (HRQoL) burden of pasireotide LAR and its relevant comparators, with data extracted from RCTs and non-randomized comparative trials.

**Table 2. PICO-based SLR inclusion and exclusion criteria.**

| **Category** | **Inclusion Criteria** | **Exclusion Criteria** |
| --- | --- | --- |
| Population | - Adult patients with acromegaly for whom surgery is not an option or has not been curative (indication for pasireotide LAR approved by US FDA) - Subpopulation of interest:   Adult patients with acromegaly for whom surgery is not an option or has not been curative and who are not adequately controlled with other medicines similar to pasireotide LAR known as ‘SSAs’ (indication for pasireotide LAR approved by EMA) | Patients with Cushing disease, corticotropin-induced adrenocortical hyperplasia, or pituitary ACTH hypersecretion |
| Interventions/ Comparators | - Pasireotide LAR - Octreotide (IV and oral) - Lanreotide - Pegvisomant - Cabergoline - Pegvisomant + octreotide - Pegvisomant + lanreotide - Radiotherapy - Standard of care/usual care/best supportive care - Placebo | Trials that are not investigating at least one of the included interventions/comparators |
| Outcomes | **Efficacy:**   - IGF-1 levels - GH levels - Biochemical control - Tumour response/volume - Acromegaly symptoms score   **Safety**   - All AEs - SAEs - Fatal AE - Specific AEs - Discontinuation due to any reason - Discontinuation due to AE   **HRQoL**   - AcroQoL score - Generic QoL score (e.g., EQ-5D, SF-36) | Outcomes other than those listed in the inclusion criteria |
| Study designs | **Interventional clinical trials including:**   - RCTs - Non-randomised comparative trials | - Single-arm trials (note: the protocol was amended in April 2021 to exclude single-arm trials) - Animal/ in-vitro studies, case reports, case series, narrative reviews, comments, editorials, expert opinion, letters, book chapters, theoretical studies - Observational studies - SLRs, meta-analyses, indirect or mixed treatment comparisons - Ad-hoc pooled analyses |
| Language | English | Journal articles and conference abstracts not available in English |
| Abbreviations: LAR, long-acting release; US FDA, United States Food and Drug Administration; SSA, somatostatin analogue; EMA, European Medicines Agency; IGF-1, insulin-like growth factor 1; AE, adverse event; QoL, quality of life; EQ-5D, EuroQol 5-Dimensions; SF-36, 36-Item Short-Form Health Survey; AcroQoL, Acromegaly Quality of Life questionnaire; ACTH, adrenocorticotropic hormone; SLR, systematic literature review | | |

Database searches conducted by two independent analysts on 10 February 2021 and retrieved 271 records. After removing 71 duplicate records, a total of 200 titles and abstracts were screened using the PICOS criteria. Full-text articles were obtained for 42 records and screened in further detail. A total of 12 trials reported in 18 publications were finally selected for inclusion in the review. Figure 1 presents the flow of studies through the search and screening process, from the initial search hits to the final set of studies included in the SLR.

**Figure 1. PRISMA flowchart diagram of study selection.**

The 12 included trials had variable trial designs. Most (n=6) were multicenter, open-label RCTs [3,4], some (n=3) were multicenter, double-blind RCTs [5–7], or (n=2) single-center trials, including one crossover RCT [8] and a double-blind RCT [9]. One trial failed to report on blinding [10]. Most trials did not report the trial phase (n=6); however, where reported, three were phase III [6,11,12], two were phase II [4,9], and one was phase IV [8]. The trials enrolled wide ranges of patients, with sample sizes ranging from 12 patients in a single-center crossover RCT [8] to 358 patients in a multicenter double-blind RCT [6]. Some trials had relative short durations between 12 and 16 weeks of treatment [5,13], with longer trials going up to six and seven months [4,6,8,10,14]. Two trials, C2305 and PAOLA had separate extension studies [15,16].

Three trials examined pasireotide LAR at different doses or compared it to octreotide and lanreotide. The first trial, C2305, was a multicenter, double-blind, phase III RCT that randomized patients to either pasireotide LAR or octreotide intramuscular (IM). Patients were required to have either undergone pituitary surgery or been contraindicated for surgery but were naïve to medical treatment. They received treatment every 28 days for 12 months [6]. C1202 was a Japanese multicenter, open-label, phase II RCT of patients with inadequately controlled acromegaly (and one with pituitary gigantism) who received various doses of pasireotide LAR (20 mg, 40 mg or 60 mg) for 12 months [4]. PAOLA was a multicenter RCT that utilized an interactive voice-web response system to randomly assign treatment-experienced patients to one of two doses of pasireotide LAR or a control (lanreotide or octreotide). C1202 had the smallest sample size, at 33 patients [4], with PAOLA and C2305 examining 198 and 358 patients, respectively [6,10].

Two trials evaluated pegvisomant [3,5]. One randomized, double-blind phase III study compared 10mg, 15mg and 20mg doses of pegvisomant with placebo in 112 patients with acromegaly of which 93 patients had undergone pituitary surgery [5]. A second randomized multicenter open label trial compared treatment with 10mg pegvisomant and 10 mg pegvisomant combined with octreotide (continuation of current dose) over a period of 40 weeks [3]. A total of 84 patients were included, of which 56 were inadequately controlled on long-acting octreotide and had previously undergone surgical treatment and/or radiotherapy. The remaining 28 patients who were adequately controlled on long-acting octreotide were assigned to a control treatment

Amongst the remaining trials examining first-generation SSAs, the trial by Andries et al. (2008) [8] treated patients with lanreotide or octreotide who, after six months, were switched to the opposite treatment without a washout period between treatments. Because of this, only the data from the first six months of the trial period (i.e., prior to washout) were extracted. Similarly, Chanson et al. (2000) [13] included patients who had been treated with lanreotide sustained release (SR) 30 mg intravenous (IV) for a mean of 26 months and then switched them over to octreotide. Ezzat et al. (1992) [7] examined 115 patients who were randomized to octreotide 100 ug for four weeks, 104 of whom were then re-randomized to octreotide 100 ug or 250 ug for six months [7]. This review focuses solely on patients from the second part of the trial. In the LANTERN trial, patients were given lanreotide autogel based on their GH and IGF-1 levels to determine the effect on tumor size. This trial enrolled two groups of patients: those who had prior surgery and those who did not. This review includes only data from the subgroup of patients who had failed previous surgery. [12]. A summary of the 12 included trials can be found in Table 3

Table 3. Overview of included studies.

| Trial ID | Place(s) of Recruitment | Trial characteristics | Population Description | Treatment Arms | Duration | Sample size (n) |
| --- | --- | --- | --- | --- | --- | --- |
| C1202 [4] | Japan | Phase II  Multicenter/Open label/RCT/Japan | Mixed population*  32 patients with acromegaly and 1 patient with pituitary gigantism who were medically naïve or inadequately controlled with somatostatin analogues or dopamine agonists. More than 80% had undergone previous surgery | - Pasireotide LAR 20, 40, 60 mg | 12 months | 33 |
| C2305 [6] | 27 countries | Phase III  Multicenter/Double-blind/RCT/Multinational | Meet FDA indication^  358 adult patients with active acromegaly who have undergone one or more pituitary surgeries but have not been treated medically, or who refuse or are contraindicated for pituitary surgery | - Pasireotide LAR 40 mg - Octreotide IM - 20 mg | 12 weeks, 26 weeks for extension | 358 |
| PAOLA (C2402) [10] | 18 countries | Phase III/Multicenter/RCT/  Multinational | Meet EMA indication~  198 patients aged 18 years or older with acromegaly who were inadequately controlled and had received 30 mg octreotide LAR or 120 mg lanreotide for 6 months or longer. Two-thirds of patients (132/198) had previously received surgery | - Pasireotide LAR 40 mg - Pasireotide 60 mg - Octreotide 30 mg or lanreotide 120 mg | 24 weeks | 198 |
| Trainer et al, 2000 [5] | UK, US | Phase III Multicenter/Double-blind/RCT/Multinational | Mixed population*  112 patients with acromegaly, of which 93 patients had undergone pituitary surgery | - Pegvisomant 10, 15, 20 mg | 12 weeks | 112 |
| Trainer et al., 2009 [3] | NR | Multicenter/Open label/RCT/Location NR | Meet EMA indication~  56 patients with acromegaly who were inadequately controlled on long-acting octreotide and who had previously undergone surgical treatment and/or radiotherapy. 28 patients who were adequately controlled on long-acting octreotide were assigned to a control group | - Pegvisomant 10 mg - Pegvisomant + octreotide (continuation of current dose) | 40 weeks | 84 |
| Andries et al., 2008 [8] | Denmark | Phase IV  Single center/Crossover RCT/Denmark | 12 acromegalic patients (7 had previously undergone surgery, 11 had received octreotide for more than 2 years) | - Lanreotide - Octreotide | 6 months | 12 |
| Bronstein et al., 2005 [9] | NR | Phase II  Single center/Double-blind/RCT/Location NR | Mixed population*  18 patients aged 18 years or older with active acromegaly who had undergone previous surgery and/or radiotherapy. 8/18 (44%) patients had undergone surgery ≥3 months before the trial | - Lanreotide 60, 90, 120 mg | 16 weeks | 18 |
| Chanson et al., 2000 [13] | France | Multicenter/Open label/RCT/Multinational | Mixed population*  125 patients with acromegaly. All patients have previously been treated with lanreotide SR 30 mg prior to trial. No patient had had surgery within 4 weeks of the trial | - Lanreotide 40 mg - Octreotide IM 20 mg | 3 months | 125 |
| Ezzat et al., 1992 [7] | US | Multicenter/Double-blind/RCT/USA | Meet FDA indication^  115 acromegalic patients, 70% of whom had persistent disease after pituitary surgery or radiotherapy | - Octreotide IM 100 ug - Octreotide IM 250 ug - Octreotide IM - Placebo | 4 weeks (part 1 of trial), 6 months (part 2 of trial) | 115 |
| Giustina et al., 2009 [11] | Italy | Phase III  Multicenter/Open label/RCT/Italy | Meet EMA indication~  28 acromegaly patients inadequately controlled despite ≥ 6 months' conventional SSA therapy, some had previous surgery, and all were medically treated | - Octreotide 60 mg (high dose) - Octreotide 30 mg (high frequency) | 24 weeks | 28 |
| Giustina et al., 2017 [14] | Italy | Multicenter/Open label/RCT/Italy | Meet EMA indication~  30 patients aged ≥18 years with biochemically active acromegaly at the time of receiving IM octreotide LAR or SC lanreotide autogel for >6 months | - Lanreotide 120 mg (high frequency) - Lanreotide 180 mg (high dose) | 24 weeks | 30 |
| LANTERN [12] | China | Phase III  Multicenter/Open label/RCT/China | Mixed population*  128 patients with active acromegaly | - Lanreotide 60, 90, 120 mg | 32 weeks | 128 |
| * Mixed patient population including those who meet the FDA indication for pasireotide LAR treatment and those who do not  ^ Meet FDA indication for pasireotide LAR treatment, i.e., adult patients with acromegaly for whom surgery is not an option or has not been curative  ~ Meet EMA indication for pasireotide LAR treatment, i.e., adult patients with acromegaly for whom surgery is not an option or has not been curative and who are not adequately controlled with other medicines similar to pasireotide LAR known as ‘SSAs’  Abbreviations: EMA, European Medicines Agency; FDA, Food and Drug Administration; IM = intramuscular; LAR, long-acting release; RCT, randomized controlled trial; SC, subcutaneous; SSA, somatostatin analogue; USA, United States of America | | | | | | |

A summary of the populations included in the 12 trials is reported in Table 4.

Table 4. Characteristics of patients in included trials.

| Patient Characteristics | High-level Overview | No. of Trials Reporting Characteristic |
| --- | --- | --- |
| Median patient age range | 46 to 53 years | 7 trials |
| Proportion of males | 33.0%–66.7% | 10 trials |
| EMA/FDA indication | -- | Mixed: 6  EMA: 4  FDA: 2 |
| Proportion of patients with previous surgery | 40.3%–100% | 10 |
| Proportion of patients that failed surgery | 40.3%–100.0% | 8 |
| Proportion of patients who were unresponsive to previous SSA | 0%–100% | 8 |
| Baseline IGF-1 | - 1.49xULN–3.1xULN - 469–784 ug/l | 9 |
| Baseline GH | 2.4–46.0 ug/l | 8 |
| Abbreviations: EMA, European Medicines Agency; FDA, Food and Drug Administration; GH, growth hormone; IGF-1, insulin-like growth factor 1; IM, intramuscular; SSA, somatostatin analogue; ULN, upper limit of normal | | |

In some trials, the patient characteristics of interest were well reported; however, others failed to examine key data such as baseline IGF-1/GH levels or previous surgery/treatment. Overall, the trials included patients of similar age, with median ages ranging from 46 to 53 years. However, other patient characteristics were vastly different. The proportion of patients who were unresponsive to previous SSAs ranged widely, from 0% of patients in LANTERN [12] and C2305 [6],18 to 100% of the patients in the PAOLA trial [10], Trainer et al. (2009) [3] and Giustina et al. (2017) [14]. Some trials also enrolled greater proportions of male patients, ranging from 33.0%–66.7%. Studies also varied regarding the EMA/FDA treatment indication: six studies had mixed indications, four had EMA indications and two had FDA indications.

Similar to the case with previous surgery, baseline IGF-1 and GH levels varied widely by trial, as did the method of reporting these data. For baseline IGF-1, some trials reported values as ug/l, while others used a threshold compared to the upper limit of normal (ULN). Additionally, baseline GH levels were presented as either mean or median. Where consistently reported, baseline IGF-1 ranged from 1.49xULN to 3.1xULN and from 469–784 ug/l. Baseline GH had a substantial range, from 2.4 to 46.0 ug/l. It should be noted, however, that the trial reporting the highest baseline IGF-1 and GH levels had a small sample size of only 18 patients [9].

Patient enrolment criteria also varied considerably across the included trials, even among those examining the same drug. The pasireotide LAR trials, C1202, C2305 and PAOLA, all enrolled a majority of patients who had undergone a previous surgery or who were contraindicated for surgery; however, C2305 required that patients be treatment naïve, while the other two trials enrolled patients who had received previous treatment [4,10]. C1202 included patients who were naïve to or inadequately controlled on previous SSAs [4], and PAOLA specifically enrolled patients who were receiving octreotide or lanreotide for at least six months prior to trial entry [10].

In the two trials examining pegvisomant, the more recent trial by Trainer et al. (2009) [3] examined different doses of the drug, while the earlier trial by Trainer et al. (2000) [5] compared pegvisomant to placebo. Both trials enrolled patients who had undergone previous surgery; Trainer et al. (2009) also required that patients received octreotide LAR for at least six months before the trial. In Trainer et al. (2000) all patients on pharmacological therapy at screening discontinued their previous treatment during a washout period and were enrolled if their IGF-1 levels were 1.3 times the ULN. This resulted in a substantial proportion in the placebo group (10%) achieving IGF-1 normalization by the end of the trial (12 weeks). In addition, patients who had received a long-acting SSA within the 12 weeks prior to trial enrolment were ineligible for the study, which may have resulted in selection bias [5].

The studies on lanreotide enrolled mixed populations of patients who had undergone previous surgery; one of the trials required that no patients receive surgery within four weeks of the trial [13], and in the other trials, the percentage of patients who had previous surgery ranged from 44% to 60% [14]. Three trials included patients who had been previously treated with SSAs [8,13,14]; Chanson et al. (2000) required that patients had been treated with lanreotide, and Giustina et al. (2017) enrolled patients previously treated with octreotide or lanreotide. For the LANTERN trial, only data from the subgroup of patients who had undergone previous surgery were included [12]. Giustina et al. (2009) examined octreotide and enrolled patients who were uncontrolled on prior SSA treatment [11]. Only data from the second part of the Ezzat et al. (1992) [7] trial were of interest to this SLR, so all patients in this group receiving octreotide had received prior treatment in the first part of the trial [7].

A summary of treatment characteristics for included trials is provided in Table 5.

Table 5. Treatment characteristics in included trials.

| Trial Name  Author, year  NCT | Treatment | Sample Size | ROA | Frequency | Duration |
| --- | --- | --- | --- | --- | --- |
| C1202 [4] | Pasireotide LAR 20 mg | 11 | NR | NR | 12 months |
|  | Pasireotide LAR 40 mg | 11 |  |  |  |
|  | Pasireotide LAR 60 mg | 11 |  |  |  |
| C2305 [6] | Pasireotide LAR 40 mg | 176 | IM | Every 28 days | 12 months; 26 months in extension |
|  | Octreotide IM 20 mg | 180 |  |  |  |
| PAOLA (C2402) [10] | Pasireotide LAR 40 mg | 63 | NR | Every 28 days | 24 weeks |
|  | Pasireotide LAR 60 mg | 63 |  |  |  |
|  | Octreotide 30 mg or lanreotide 120 mg | 66 |  |  |  |
| Giustina et al., 2017 [14] | Lanreotide 120 mg (high frequency) | 14 | NR | Every 21 days | 24 weeks |
|  | Lanreotide 180 mg (high dose) | 15 |  | Every 28 days |  |
| LANTERN [12] | Lanreotide autogel 60 mg, 90 mg or 120 mg | 39 | SC | Every 4 weeks | 32 weeks |
| Andries et al., 2008 [8] | Lanreotide | 6 | SC | Every 28 days | 6 months |
| Bronstein et al., 2005 [9] | Lanreotide 60 mg | 6 | SC | Every 28 days | 16 weeks |
|  | Lanreotide 90 mg | 6 |  |  |  |
|  | Lanreotide 120 mg | 6 |  |  |  |
| Chanson et al., 2000 [13] | Lanreotide 30 mg | 21 | IM | Every 10 days or every 14 days | 3 months |
|  | Octreotide IM 20 mg | 86 |  | Every month |  |
| Ezzat et al., 1992 [7] | Placebo | 55 | SC | Every 8 hours | 6 months |
|  | Octreotide IM 100 ug | 60 |  |  |  |
| Giustina et al., 2009 [11] | Octreotide IM 60 mg (high dose) | 11 | IM | Every 28 days | 24 weeks |
|  | Octreotide IM 30 mg (high frequency) | 15 |  | Every 21 days |  |
| Trainer et al., 2000 [5] | Placebo | 31 | SC | Once daily | 12 weeks |
|  | Pegvisomant 10 mg | 26 |  |  |  |
|  | Pegvisomant 15 mg | 26 |  |  |  |
|  | Pegvisomant 20 mg | 28 |  |  |  |
| Trainer et al., 2009 [3] | Pegvisomant 10 mg | 25 | SC | Once daily | 40 weeks |
|  | Pegvisomant 10 mg + cont. of octreotide | 26 |  |  |  |
| Abbreviations: IM, intramuscular; LAR, long-acting release; NR, not reported; RCT, randomized controlled trial; ROA, route of administration; SC, subcutaneous; SLR, systematic literature review | | | | | |

Among the included trials, lanreotide was the most commonly evaluated treatment (n=5) [8,9,12–14], followed by pasireotide LAR (n=3) [4,6,10]. Two trials each examined octreotide [7,11] and pegvisomant as the main interventions [3,5].

In the three trials examining pasireotide LAR, it was compared at various doses or to octreotide or lanreotide. The C1202 trial examined pasireotide LAR at doses of 20 mg, 40 mg and 60 mg for 12 months among a sample of 33 patients [4]. Anyone who received the 20 mg or 40 mg doses could have their doses increased to 60 mg; also, doses could be decreased if deemed necessary by the trial investigators. In C2305, patients were randomized to pasireotide LAR 40 mg or octreotide 20 mg IM every 28 days for 12 months [6]. Patients could have their doses increased up to 60 mg by three months or seven months, depending upon their biochemical control. In the PAOLA trial, patients were randomized to pasireotide LAR 40 mg, pasireotide LAR 60 mg, octreotide 30 mg or lanreotide 120 mg autogel IM for 24 weeks; if tolerability issues arose, patients could reduce their dose.10 Each trial allowed concomitant diabetes medication during the treatment period.

Two trials by Trainer et al. (2000, 2009) [3,5] compared pegvisomant monotherapy to combination treatment with pegvisomant and octreotide as well as to placebo. In the earlier trial, patients received pegvisomant 10 mg, 15 mg, 20 mg or placebo as once-daily subcutaneous (SC) injections for 12 weeks [5]. In the second trial, patients received either pegvisomant 10 mg or pegvisomant 10 mg plus their current dose of octreotide as SC injections every day for 40 weeks. Doses of pegvisomant were adjusted according to patient tolerability by 5 mg every eight weeks to a maximum dose of 30 mg or a minimum dose of 5 mg [3].

Five trials examined lanreotide, three of which examined various doses of lanreotide [8,9,14] and two of which compared lanreotide to octreotide [12,13]. In Andries et al. (2008) patients received different doses of lanreotide dependent upon their previous dose of octreotide. Trial investigators used a conversion algorithm to determine the dose of lanreotide each patient received subcutaneously every 28 days for six months [8]. Similarly, patients in the trial by Bronstein et al. (2005) were randomized to lanreotide 60 mg, 90 mg or 120 mg every 28 days for 16 weeks; half of the patients were also treated with concomitant octreotide [9]. Giustina et al. (2017) reported on high-frequency (120 mg every 21 days) and high-dose (160 mg every 28 days) regimens of lanreotide for 24 weeks [14]. In Chanson et al. (2000), all patients were treated with lanreotide 30 mg every 10 days or every day for three months and then given octreotide 20 mg once a month for three months. Octreotide doses could be adjusted based on patients’ GH levels at the end of the first three months [13]. In LANTERN, patients received lanreotide autogel 60 mg, 90 mg or 120 mg based on their GH and IGF-1 levels; each dose was given SC every four weeks for 32 total weeks [12].

Ezzat et al. (1992) and Giustina et al. (2009) both reported on octreotide treatment among patients with acromegaly [7,11]. In the second part of the Ezzat trial, patients received either octreotide IM 100 ug or 250 ug as an SC injection every eight hours for six months [7]. Patients enrolled in the trial by Giustina et al. (2017) received either high-dose octreotide (60 mg every 28 days) or high-frequency octreotide (30 mg every 21 days) intramuscularly for 24 weeks [14].

A summary of outcomes of interest presented in included trials is presented in Table 6.

Table 6. Outcomes of interest in included trials.

| Trial Name  Author, year  NCT | Biochemical Control | GH Level | IGF-1 Level | Tumor Response | AEs | Acromegaly Symptom Score | HRQoL | Primary outcome |
| --- | --- | --- | --- | --- | --- | --- | --- | --- |
| **Pasireotide LAR** | | | | | | | |  |
| C1202 [4] | YES | YES | YES | YES | YES | YES | NO | Proportion of all patients who achieved biochemical control, i.e., mean GH <2.5 μg/L and age- and sex-adjusted normal IGF-1 at month 3 |
| C2305 [6] | YES | NO | NO | YES | YES | YES | YES | Proportion of patients achieving GH <2.5 µg/L and normal IGF-1 for age and sex at month 12 |
| PAOLA (C2402) [10] | YES | YES | YES | YES | YES | YES | YES | Proportion of patients achieving biochemical control (mean GH concentration <2.5μg/L and normalised IGF-1 concentration [between the upper and lower limits of normal]) at 24 weeks |
| **Pegvisomant** | | | | | | | |  |
| Trainer et al., 2000 [5] | YES | YES | YES | NO | YES | YES | NO | Percentage change in the serum IGF-1 concentration from baseline |
| Trainer et al., 2009 [3] | YES | YES | NO | YES | YES | YES | YES | Number of treatment-emergent AEs in the safety population (all patients receiving at least one dose of study drug) |
| **Lanreotide** | | | | | | | |  |
| Andries et al., 2008 [8] | YES | NO | YES | NO | YES | YES | NO | GH and IGF-1 levels after 4, 6, 10, 12 months therapy |
| Bronstein et al., 2005 [9] | YES | NO | YES | NO |  | NO | NO | Pharmacokinetic profile of three dose levels of lanreotide autogel |
| Chanson et al., 2000 [13] |  | NO |  | NO | YES | NO | NO | Mean GH and IGF-1 levels and proportion of patients with normal IGF-1 levels |
| Giustina et al., 2017 [14] | YES | NO | YES | NO | YES | NO | NO | Proportion of patients with normalisation of serum IGF-1 (≤1.2 x ULN) and decrease of random GH <1 µg/L at the end of follow-up (24 weeks of treatment) |
| LANTERN [12] | YES | YES | YES | YES | YES | NO | NO | Mean change-from baseline in age-adjusted IGF-1 level (expressed as standard deviation score) at the end-of-study/early withdrawal visit |
| **Octreotide** | | | | | | | |  |
| Ezzat et al., 1992 [7] | YES | NO | NO | YES | NO | NO | NO | GH and IGI-1 levels |
| Giustina et al., 2009 [11] | YES | YES | YES | YES | YES | NO | NO | Mean change in IGF-1 and GH serum concentrations from baseline to week 24. |
| **Abbreviations**: AE, adverse event; GH, growth hormone; HRQoL, health-related quality of life; IGF-1, insulin-like growth factor-1; LAR, long-acting release | | | | | | | | |

# References

[1]	Cumpston M, Li T, Page MJ, et al. Updated guidance for trusted systematic reviews: a new edition of the Cochrane Handbook for Systematic Reviews of Interventions. Cochrane Database Syst Rev 2019;10:ED000142. https://doi.org/10.1002/14651858.ED000142.

[2]	Chaimani A, Caldwell DM, Li T, et al. Chapter 11: Undertaking network meta-analyses. In: Higgins JPT, Thomas J, Chandler J, et al., editors. Cochrane Handb. Syst. Rev. Interv. Version 65, Cochrane; 2024.

[3]	Trainer PJ, Ezzat S, D’Souza GA, et al. A randomized, controlled, multicentre trial comparing pegvisomant alone with combination therapy of pegvisomant and long-acting octreotide in patients with acromegaly. Clin Endocrinol (Oxf) 2009;71:549–57. https://doi.org/10.1111/j.1365-2265.2009.03620.x.

[4]	Tahara S, Murakami M, Kaneko T, et al. Efficacy and safety of long-acting pasireotide in Japanese patients with acromegaly or pituitary gigantism: results from a multicenter, open-label, randomized, phase 2 study. Endocr J 2017;64:735–47. https://doi.org/10.1507/endocrj.EJ16-0624.

[5]	Trainer PJ, Drake WM, Katznelson L, et al. Treatment of acromegaly with the growth hormone-receptor antagonist pegvisomant. N Engl J Med 2000;342:1171–7. https://doi.org/10.1056/NEJM200004203421604.

[6]	Colao A, Bronstein MD, Freda P, et al. Pasireotide versus octreotide in acromegaly: a head-to-head superiority study. J Clin Endocrinol Metab 2014;99:791–9. https://doi.org/10.1210/jc.2013-2480.

[7]	Ezzat S, Snyder PJ, Young WF, et al. Octreotide treatment of acromegaly. A randomized, multicenter study. Ann Intern Med 1992;117:711–8. https://doi.org/10.7326/0003-4819-117-9-711.

[8]	Andries M, Glintborg D, Kvistborg A, et al. A 12-month randomized crossover study on the effects of lanreotide Autogel and octreotide long-acting repeatable on GH and IGF-l in patients with acromegaly. Clin Endocrinol Oxf 2008;68:473–80. https://doi.org/10.1111/j.1365-2265.2007.03067.x.

[9]	Bronstein M, Musolino N, Jallad R, et al. Pharmacokinetic profile of lanreotide Autogel in patients with acromegaly after four deep subcutaneous injections of 60, 90 or 120 mg every 28 days. Clin Endocrinol (Oxf) 2005;63:514–9. https://doi.org/10.1111/j.1365-2265.2005.02372.x.

[10]	Gadelha MR, Bronstein MD, Brue T, et al. Pasireotide versus continued treatment with octreotide or lanreotide in patients with inadequately controlled acromegaly (PAOLA): a randomised, phase 3 trial. Lancet Diabetes Endocrinol 2014;2:875–84. https://doi.org/10.1016/S2213-8587(14)70169-X.

[11]	Giustina A, Bonadonna S, Bugari G, et al. High-dose intramuscular octreotide in patients with acromegaly inadequately controlled on conventional somatostatin analogue therapy: a randomised controlled trial. Eur J Endocrinol 2009;161:331–8. https://doi.org/10.1530/EJE-09-0372.

[12]	An Z, Lei T, Duan L, et al. Efficacy and safety of lanreotide autogel compared with lanreotide 40 mg prolonged release in Chinese patients with active acromegaly: results from a phase 3, prospective, randomized, and open-label study (LANTERN). BMC Endocr Disord 2020;20:57. https://doi.org/10.1186/s12902-020-0524-7.

[13]	Chanson P, Boerlin V, Ajzenberg C, et al. Comparison of octreotide acetate LAR and lanreotide SR in patients with acromegaly. Clin Endocrinol (Oxf) 2000;53:577–86. https://doi.org/10.1046/j.1365-2265.2000.01134.x.

[14]	Giustina A, Mazziotti G, Cannavo S, et al. High-Dose and High-Frequency Lanreotide Autogel in Acromegaly: A Randomized, Multicenter Study. J Clin Endocrinol Metab 2017;102:2454–64. https://doi.org/10.1210/jc.2017-00142.

[15]	Sheppard M, Bronstein MD, Freda P, et al. Pasireotide LAR maintains inhibition of GH and IGF-1 in patients with acromegaly for up to 25 months: results from the blinded extension phase of a randomized, double-blind, multicenter, Phase III study. Pituitary 2015;18:385–94. https://doi.org/10.1007/s11102-014-0585-6.

[16]	Colao A, Bronstein MD, Brue T, et al. Pasireotide for acromegaly: long-term outcomes from an extension to the Phase III PAOLA study. Eur J Endocrinol 2020;182:583. https://doi.org/10.1530/EJE-19-0762.
