# Supplementary material for: Cost−effectiveness of pasireotide as second−line therapy for adult acromegaly patients from the perspective of the Brazilian Unified Health System
Source: Front Endocrinol (Lausanne). 2026 Aug 5;17:1771580. doi: 10.3389/fendo.2026.1771580 (PMC13487751; doi:10.3389/fendo.2026.1771580)
Supplement: Supplementary file 2 [file SupplementaryFile2.docx]

# Indirect treatment comparison details

A systematic literature review was conducted according to NICE standards [1] to identify all relevant published evidence on drug interventions for the treatment of acromegaly (including RCTs and observational studies/registries), and relevant data from the selected studies was subsequently retrieved (see online resource 1). Selected studies were assessed for the feasibility of indirect comparisons based on study design characteristics, populations included, and outcomes assessed. To be eligible for inclusion in the ITC analysis, studies had to fulfil the following eligibility criteria:

1. Study design: Randomized Controlled Trial
2. Have common comparators for the construction of a network diagram
3. Include homogeneous populations
4. Outcomes assessed: GH control, IGF-1 normalization, biochemical response

Indirect comparisons of the selected studies were performed following Bucher’s fixed effects model [2]. The choice of the fixed effects model vs. a random effects model was based on the low number of studies identified. All analyses performed hereafter compared treatments using the Odds Ratio outcome framework.

For this analysis, first generation somatostatin receptor ligands (FGSRLs) octreotide and lanreotide have been considered equivalent in terms of efficacy, under the “SSA” [3].

Table 7. Key characteristics of studies selected for indirect comparison.

| Study | FU (w) | Population | N | Tx Arms (n) – ITT | Mean Age (yrs) | M (%) | S (%) | RT (%) | MT (%) |
| --- | --- | --- | --- | --- | --- | --- | --- | --- | --- |
| C2402 (PAOLA) [4] | 24 | Patients inadequately controlled with FGSLR treatment | 198 | PAS LAR 40mg (65)  PAS LAR 60mg (65)  FGSLRs (68) | 42.9  45.8  46.2 | 41.5  46.2  44.1 | 76.9  63.1  60.3 | 3.1  4.6  7.4 | FGSLR: 100 |
| Trainer et al., 2000 [5] | 12 | Mixed population (naïve, medically naïve and previously medically treated) | 112 | PEG 10mg (26)  PEG 15mg (26)  PEG 20mg (28)  Placebo (32) | 47.0  46.0  48.0  50.0 | 58  54  54  59 | 85  85  82  81 | 42  62  66  58 | FGSLR: 72  DA: 49 |
| Trainer et al., 2009 [6] | 40 | Patients inadequately controlled with SSA treatment | 84 | PEG mono (25)  PEG +FGSLR (26)  OCT LAR (27) | 49.0  40.0  45.0 | 60  65  44 | NR | NR | OCT LAR: 100 |
| Abbreviations: Tx, treatment; ITT= intent-to-treat population; FU, study follow-up; M, male; S, previous surgery; RT, previous radiotherapy; MT, previous medical treatment; DB, double blind; OL, open label; RCT, randomized clinical trial; FGSLR, first generation somatostatin receptor ligands; DA, dopamine agonist; OCT LAR= octreotide Long-acting release; PAS LAR, pasireotide long-acting release; PEG, pegvisomant; NR= not reported | | | | | | | | | |

An analysis using a Bucher fixed-effect model was possible using the proportion of patients achieving IGF-1 normalization after treatment using odds ratios (ORs). The following assumptions were made:

1. It was assumed that baseline differences in patient characteristics across the three trials were mitigated by the ITC employing relative effectiveness established via common comparators [2,7]. . The analysis of patients’ baseline characteristics from the studies showed heterogeneity in terms of some baseline characteristics, including baseline GH levels and proportions of patients with previous RT treatment, which are potential prognostic factors [8–10]. Notably, in the PAOLA study [4] the baseline GH levels were 17.6 µg/L and 12.1 µg/L in the PAS LAR 40 mg and PAS LAR 60 mg arms, respectively. In contrast, GH levels in the pegvisomant arms varied between 3.0 µg/L and 4.2 µg/L in Trainer et al. (2009) [6] and between 7.8 µg/L and 11.5 µg/L in Trainer et al. (2000) [5], indicating that patients had less severe acromegaly than those in the PAOLA study. Only 3–5% of PAS LAR patients had previously received RT, compared to 42–66% in Trainer et al. (2000) [5] (no information on previous RT in Trainer et al., 2009 [6]). Unfortunately, it was not possible to compare baseline IGF-1 levels between the PAS LAR and pegvisomant studies (in PAOLA, these were reported as multiples of ULN; in Trainer et al.(2000) [5] and Trainer et al. (2009) [6], mean absolute values were reported).
2. In the PAOLA study, no patients allocated to the active control arm presented normalized IGF-1 levels at the end of the study. To estimate the OR for the comparison against both pasireotide LAR groups, the event rate was artificially increased by adding 0.5 to all trial arms, as previously reported in the literature [11].
3. Based on Colao et al. (2011) [8], octreotide and lanreotide were considered equivalent in terms of IGF-1 efficacy, and they were combined as ‘FGSRLs’.
4. As the duration of the three included studies ranged between 12 weeks and 40 weeks, it was assumed that patients had achieved stable response at the end of the respective follow-up periods. This assumption was supported by evidence from the studies. Trainer et al. (2000) [5] reported stable average serum IGF-1 concentrations after two weeks of pegvisomant treatment, whereas Trainer et al. (2009) [6] reported a significant decrease in the mean serum IGF-1 levels at week 8 and a slow decline until week 40. PAOLA (C2402) [4] reported similar mean values of standardized IGF-1 levels at 12 and 24 weeks (end of core trial).
5. Drug regimens used in the studies included in the analyses were heterogeneous, but the analysis assumes equivalency. Trainer et al. (2009) [6] did not establish fixed doses for both treatment arms (pegvisomant monotherapy and pegvisomant plus SSA); for Trainer et al. (2000) [5] the median pegvisomant doses (20 mg/day monotherapy group and 15 mg/day combination therapy group) have been assumed.
6. An assumption on the similarity of the control arms of the PAOLA study (FGSLRs) [4] and the Trainer et al. (2000) [6] study (placebo) was required. As a larger efficacy difference should be expected for a comparison with placebo than for a comparison with FGSLRs, this assumption potentially favors pegvisomant. However, this was the only solution to establish relative efficacy between PAS LAR and pegvisomant.

The assumptions around the comparability of patient populations, drug dosage and timing of outcomes are likely to limit the robustness of the ITC.

An evidence network used to assess the comparative efficacy of pasireotide LAR against comparators is presented in Figure 1

Figure 2. Indirect comparison network.

The data included in the ITC analyses are presented in Table 2.

Table 8. Data included in the ITC analyses.

| Trial ID y | Treatment Arms (n) – ITT | Proportion achieving IGF-1 normalization  n (%) | OR (95% CI) |
| --- | --- | --- | --- |
| PAOLA (C2402) [4] | Pasireotide LAR 40 mg (n=65)  Pasireotide LAR 60 mg (n=65)  SSAs (octreotide 30 mg or lanreotide 120 mg) (n=68) | 16/65 (25%)  17/65 (26%)  0/68 (0%) | Pasireotide LAR 40 mg vs. FGSRLs: 45.7 (2.7; 780.1)  Pasireotide LAR 60 mg vs. FGSRLs: 49.4 (2.9; 841.2)* |
| Trainer et al., 2000 [5] | Pegvisomant 10 mg (n=26)  Pegvisomant 15 mg (n=26)  Pegvisomant 20 mg (n=28)  Placebo (n=32) | 10/26 (38%)  18/26 (69%)  23/28 (82%)  3/32 (9%) | Pegvisomant 10 mg vs. Placebo: 6.04 (1.45; 25.17)  Pegvisomant 15 mg vs. Placebo: 21.75 (5.09; 92.94)  Pegvisomant 20 mg vs. Placebo: 44.67 (9.60; 205.92) |
| Trainer et al., 2009 [6] | Pegvisomant 10 mg (n=25)  Pegvisomant 15 mg + SSA (continuation current octreotide dose) (n=26)  Octreotide LAR (n=27) | 14/25 (56%)  16/26 (62%)  Not reported | Pegvisomant 15 mg + SSA (continuation current octreotide dose) vs. Pegvisomant 10 mg: 1.26 (0.41; 3.84) |
| Abbreviations: CI, confidence interval; IGF-1, insulin-like growth factor 1; ITT, intention-to-treat; LAR, long-acting release; OR, odds ratio; FGSRLs first generation somatostatin receptor ligands | | | |

The results from the ITC analyses are reported in Online Resource 3.

# Derivation of transition probabilities for the economic model

Response data extracted from the clinical trials were not used directly in the cost-effectiveness model. Instead, they were transformed into treatment-specific probabilities using the odds ratios obtained from ITC. This process was performed in four steps. First, response data were extracted from the eligible clinical trials included in the ITC. Second, an ITC using Bucher’s fixed-effect method was conducted to estimate relative treatment effects for pegvisomant-based regimens versus pasireotide LAR, expressed as odds ratios for IGF-1 normalization. Third, these relative effects were anchored to the absolute probabilities observed for pasireotide LAR in PAOLA, which served as the reference efficacy profile in the model. Fourth, the resulting estimated absolute probabilities for pegvisomant-based regimens were mapped to the model health states used in Table 3.

In the model, full control (FC) was defined as IGF-1 ≤1×ULN and partial control (PC) as at least a 50% decrease in IGF-1. Because PC was not directly reported in PAOLA, patients who had not achieved FC at the first assessment (after 3 months) but achieved FC by the second assessment (after 6 months) were assumed to be in PC at the first assessment. The same framework was subsequently applied to pegvisomant-based regimens after converting the ITC results into absolute probabilities.

References

[1]	National Institute for Health and Care Excellence. NICE health technology evaluations: the manual. Process Methods PMG36 2022.

[2]	Bucher HC, Guyatt GH, Griffith LE, et al. The results of direct and indirect treatment comparisons in meta-analysis of randomized controlled trials. J Clin Epidemiol 1997;50:683–91. https://doi.org/10.1016/s0895-4356(97)00049-8.

[3]	Murray RD, Melmed S. A critical analysis of clinically available somatostatin analog formulations for therapy of acromegaly. J Clin Endocrinol Metab 2008;93:2957–68. https://doi.org/10.1210/jc.2008-0027.

[4]	Gadelha MR, Bronstein MD, Brue T, et al. Pasireotide versus continued treatment with octreotide or lanreotide in patients with inadequately controlled acromegaly (PAOLA): a randomised, phase 3 trial. Lancet Diabetes Endocrinol 2014;2:875–84. https://doi.org/10.1016/S2213-8587(14)70169-X.

[5]	Trainer PJ, Drake WM, Katznelson L, et al. Treatment of acromegaly with the growth hormone-receptor antagonist pegvisomant. N Engl J Med 2000;342:1171–7. https://doi.org/10.1056/NEJM200004203421604.

[6]	Trainer PJ, Ezzat S, D’Souza GA, et al. A randomized, controlled, multicentre trial comparing pegvisomant alone with combination therapy of pegvisomant and long-acting octreotide in patients with acromegaly. Clin Endocrinol (Oxf) 2009;71:549–57. https://doi.org/10.1111/j.1365-2265.2009.03620.x.

[7]	Chaimani A, Caldwell DM, Li T, et al. Chapter 11: Undertaking network meta-analyses. In: Higgins JPT, Thomas J, Chandler J, et al., editors. Cochrane Handb. Syst. Rev. Interv. Version 65, Cochrane; 2024.

[8]	Colao A, Pivonello R, Grasso LFS, et al. Determinants of cardiac disease in newly diagnosed patients with acromegaly: results of a 10 year survey study. Eur J Endocrinol 2011;165:713–21. https://doi.org/10.1530/EJE-11-0408.

[9]	Araujo-Castro M, Biagetti B, Menendez Torre E, et al. Pegvisomant and pasireotide in PRL and GH co-secreting vs GH-secreting Pit-NETs. Endocr Relat Cancer 2024;31. https://doi.org/10.1530/ERC-24-0043.

[10]	Howlett TA, Willis D, Walker G, et al. Control of growth hormone and IGF1 in patients with acromegaly in the UK: responses to medical treatment with somatostatin analogues and dopamine agonists. Clin Endocrinol (Oxf) 2013;79:689–99. https://doi.org/10.1111/cen.12207.

[11]	Higgins JPT, Thomas J, Chandler J, Cumpston M, Li T, Page MJ, Welch VA (editors). Cochrane Handbook for Systematic Reviews of Interventions version 6.5 (updated August 2024). Cochrane, 2024. n.d.
