# Supplementary material for: Cost−effectiveness of pasireotide as second−line therapy for adult acromegaly patients from the perspective of the Brazilian Unified Health System
Source: Front Endocrinol (Lausanne). 2026 Aug 5;17:1771580. doi: 10.3389/fendo.2026.1771580 (PMC13487751; doi:10.3389/fendo.2026.1771580)
Supplement: Supplementary file 3 [file SupplementaryFile3.docx]

# Model inputs

# 1. Efficacy input

Table 9. Efficacy input based on indirect treatment comparison (for details see Online Resources 2).

| Comparison | OR (95% CI) |
| --- | --- |
| Alternative treatment vs PAS LAR 40 mg | |
| Pegvisomant 10 mg/day | 0.132 (0.006; 3.171) |
| Pegvisomant 15 mg/day | 0.476 (0.020; 11.547) |
| Pegvisomant 20 mg/day | 0.974 (0.039; 24.506) |
| Alternative treatment vs PAS LAR 60 mg | |
| Pegvisomant 15 mg/day + FGSRLs (continuation current octreotide dose) | 1.224 (0.040; 37.140) |
| Pegvisomant 10 mg/day | 0.122 (0.005; 2.919) |
| Pegvisomant 15 mg/day | 0.440 (0.018; 10.629) |
| Pegvisomant 20 mg/day | 0.900 (0.036; 22.560) |
| Pegvisomant 15 mg/day + FGSRLs (continuation current octreotide dose) | 1.131 (0.037; 34.235) |

Table 10. Efficacy input based on real world evidence.

| Study and characteristic | Population | Treatment arms | Treatment duration | IGF-1 normalization rate (95% CI) |
| --- | --- | --- | --- | --- |
| Biagetti et al., 2025 [1],  a systematic review and meta-analysis of real-world evidence studies | Twelve studies (11 independent samples) comprising 409 acromegaly patients resistant to FGSRLs | PAS LAR monotherapy* | Ranging from 6 to 58 months | 0.58 (0.48–0.67) in random effect model  0.56 (0.51-0.61) in common effect model |
| Leonart et al., 2019 [2], systematic review and meta-analysis of observational longitudinal studies | Fourteen studies (8 independent samples for PEG regimens**) comprising 1288 acromegaly patients resistant to FGSLR on PEG regimens | PEG regimens (monotherapy and combinations) | PEG regimens: 3–96 months | 0.61 (0.52–0.69) in common effect model |
| *All but one (Urbani et al., 2024) [3] used PAS LAR as monotherapy  **Pegvisomant monotherapy or combination therapies  Abbreviations: CI – confidence interval; FGSRL – first-generation somatostatin receptor ligand; IGF-1 – insulin-like growth factor 1; PAS LAR – pasireotide long-acting release; PEG – pegvisomant. | | | | |

# 2. Safety Input

Table 11. Adverse events probabilities.

| **Adverse Event** | **PAS** | **OCT** | **LAN*** | **PEGV** | **PEG+OCT**** | **PEG+LAN** | **CAB** |
| --- | --- | --- | --- | --- | --- | --- | --- |
| Abdominal distension | 0.00% | 0.00% | 6.02% | 0.00% | 0.00% | 0.00% | 0.00% |
| Abdominal pain | 1.60% | 0.00% | 8.49% | 0.00% | 1.09% | 1.09% | 1.70% |
| Blood glucose increased | 0.00% | 0.00% | 1.92% | 0.00% | 2.19% | 2.19% | 0.00% |
| Cholelithiasis | 2.47% | 2.63% | 6.30% | 0.00% | 0.00% | 0.00% | 0.00% |
| Decreased appetite | 0.00% | 0.00% | 2.74% | 0.00% | 0.00% | 0.00% | 0.00% |
| Diarrhea | 0.00% | 1.59% | 1.59% | 3.05% | 2.19% | 2.19% | 1.70% |
| Gastrointestinal disorders | 0.00% | 0.00% | 0.00% | 0.00% | 3.83% | 3.83% | 3.39% |
| Headache | 0.00% | 0.00% | 2.74% | 2.29% | 0.00% | 0.00% | 2.26% |
| Hyperglycemia | 9.60% | 0.00% | 3.28% | 0.00% | 0.00% | 0.00% | 0.00% |
| Infections and infestations | 0.00% | 0.00% | 0.00% | 0.00% | 3.28% | 3.28% | 2.26% |
| Musculoskeletal and connective tissue disorders | 0.00% | 0.00% | 0.00% | 0.00% | 0.55% | 0.55% | 4.52% |
| Nausea | 0.00% | 0.00% | 1.92% | 3.05% | 0.00% | 0.00% | 0.00% |
| Nervous system disorders | 0.00% | 0.00% | 0.00% | 0.00% | 1.09% | 1.09% | 2.83% |
| Pain | 0.00% | 0.00% | 0.00% | 3.05% | 0.00% | 0.00% | 0.00% |
| Sinus bradycardia | 0.00% | 0.00% | 2.46% | 0.00% | 0.00% | 0.00% | 0.00% |
| Tumor growth | - | - | - | 0.50% | 0.72% | 0.72% | 0.00% |
| Upper respiratory tract infection | 0.00% | 0.00% | 5.75% | 3.82% | 0.00% | 0.00% | 2.26% |
| N | 125 | 66 | 66 | 28 | 32 | 32 | 31 |
| Treatment exposure (weeks) | 24.00 | 24.00 | 24.00 | 12.00 | 17.39 | 17.39 | 17.39 |
| *Weight decrease was assumed not to have impact on quality of life and was excluded from analysis  **Adverse event called “investigations” was excluded from analysis due to unclear nature  Abbreviations: AE, adverse event; CAB, cabergoline; LAN, lanreotide; PAS, pasireotide; PEGV, pegvisomant; OCT, octreotide. | | | | | | | |

Table 12. Adverse events data sources.

| **Treatment** | **Related publication/trial** | **AE definition** | **Duration of treatment exposure** | **N evaluated** |
| --- | --- | --- | --- | --- |
| Pasireotide | PAOLA [4] | AEs (more than 5% in any all grade column) | 24 weeks | 165 |
| Octreotide max dose | PAOLA [4] | AEs (more than 5% in any all grade column) | 24 weeks | 66 |
| Lanreotide max dose | PAOLA [4] | AEs (more than 5% in any all grade column) | 24 weeks | 66 |
| Pegvisomant* | Trainer et al., 2000 [5] | AEs that occurred in at least 10% of patients | 12 weeks | 28 |
| Pegvisomant + Octreotide | Colao et al., 2019 [6] | AEs by system organ class includes AEs occurring in ≥ 5% of patients overall | 4 months | 31 |
| Pegvisomant + Lanreotide | NA | Assumed to equal to PEG+OCT | Assumed to equal to PEG+OCT | NA |
| Tumor growth AE applied to Pegvisomant-based treatments | Leonart et al, 2019  (Systematic review and meta-analysis) [2] | NR | 3.5 years** | 1,221 |
| *Note: From pegvisomant 10/15/20 mg-dose related AE incidence, CEM applies 20 mg dose specific ones  ** Calculated as weighted average of mean treatment duration of studies (2 studies included in the meta-analysis that do not report treatment duration (Basalvibaso et al., 2010 [7] and Haliloglu et al., 2016 [8]) are not considered in the calculation)  Abbreviations: AE = adverse event; NA = not available; NR = not reported; OCT = octreotide; PEG = pegvisomant | | | | |

Table 13. Probabilities of discontinuation due to AEs during the initial model phase.

| **Second-line Treatment** | **Three-month probability** | **N** | **Source** |
| --- | --- | --- | --- |
| Pasireotide* | 2.62% | 63 | PAOLA [4] |
| Octreotide max dose | 0.67% | 38 | Trainer et al., 2009 [9] |
| Lanreotide max dose | 10.56% | 10 | Andries et al., 2008 [10] |
| Pegvisomant | 5.53% | 25 | Trainer et al., 2009 [9] |
| Pegvisomant + Octreotide | 5.30% | 26 | Trainer et al., 2009 [9] |
| Pegvisomant + Lanreotide | 10.56% | -- | Assumption: maximum of pegvisomant and lanreotide values |

# 3. Utility and disutility input

Table 14. Utility decrements.

| **Adverse event** | **Utility decrement** | **SE** | **Duration (months)*** | **Source of utility** | **Notes** |
| --- | --- | --- | --- | --- | --- |
| **Utility decrements for AEs** | | | | | |
| Abdominal distension | 0.1100 | 0.0110 | 0.50 | Shiroiwa et al., 2021 [11] | Assumed to equal to abdominal pain or stomachache |
| Abdominal pain | 0.1100 | 0.0110 | 0.50 | Shiroiwa et al., 2021 [11] | Abdominal pain or stomachache |
| Blood glucose increased | 0.0604 | 0.0001 | 0.50 | Shiroiwa et al., 2021 [11] | Assumed to equal to hyperglycemia |
| Cholelithiasis | 0.1100 | 0.0110 | 0.50 | Shiroiwa et al., 2021 [11] | Assumed to equal to abdominal pain or stomachache  SE is assumed to be 10% of mean |
| Decreased appetite | 0.1360 | 0.0136 | 0.50 | Shiroiwa et al., 2021 [11] | Lack of appetite disutility is applied  SE is assumed to be 10% of mean |
| Diabetes mellitus | 0.1442 | 0.0001 | 0.50 | Whittington et al., 2021 [12] | Not applied in case comorbidity disutilities are already accounting for it |
| Diarrhea | 0.0660 | 0.0066 | 0.50 | Shiroiwa et al., 2021 [11] | SE is assumed to be 10% of mean |
| Gastrointestinal disorders | 0.1100 | 0.0110 | 0.50 | Shiroiwa et al., 2021 [11] | Assumed to equal to abdominal pain or stomachache  SE is assumed to be 10% of mean |
| Headache | 0.0930 | 0.0093 | 0.50 | Shiroiwa et al., 2021 [11] | SE is assumed to be 10% of mean |
| Hyperglycemia | 0.0604 | 0.0060 | 0.50 | Harris et al., 2014 [13] | Assumed to equal to hypoglycemia disutility. Estimate as the average of daytime (0.0616) and nighttime (0.0592) severe hypoglycemic events  SE is assumed to be 10% of mean |
| Infections and infestations | 0.0660 | 0.0066 | 0.50 | Shiroiwa et al., 2021 [11] | Assumed equal to upper respiratory infection  SE is assumed to be 10% of mean |
| Musculoskeletal and connective tissue disorders | 0.1330 | 0.0133 | 0.50 | Shiroiwa et al., 2021 [11] | Assumed to equal to average of back pain and arthritic pain, 0.112 and 0.154, respectively  SE is assumed to be 10% of mean |
| Nausea | 0.0980 | 0.0098 | 0.50 | Shiroiwa et al., 2021 [11] | Assumed to equal to dizziness  SE is assumed to be 10% of mean |
| Nervous system disorders | 0.1525 | 0.0153 | 0.50 | Shiroiwa et al., 2021 [11] | Average of sleeplessness and irritability, 0.165 and 0.14, respectively  SE is assumed to be 10% of mean |
| Pain | 0.1100 | 0.0110 | 0.50 | Shiroiwa et al., 2021 [11] | Assumed to equal to abdominal pain or stomachache  SE is assumed to be 10% of mean |
| Sinus bradycardia | 0.0980 | 0.0098 | 0.50 | Shiroiwa et al., 2021 [11] | Assumed to equal to dizziness  SE is assumed to be 10% of mean |
| Tumor growth | 0.1525 | 0.0153 | 0.50 | - | Assumed to be maximum of all available utilities (nervous system disorders)  SE is assumed to be 10% of mean |
| Upper respiratory tract infection | 0.0660 | 0.0066 | 0.50 | Shiroiwa et al., 2021 [11] | Assume equal to cough  SE is assumed to be 10% of mean |
| **Utility decrements for comorbidities** | | | | | |
| Moderate hypertension | 0.1282 | 0.0001 | NA | Whittington et al., 2021 [12] | Hypertension disutility is applied |
| Severe hypertension | 0.1282 | 0.0001 | NA | Whittington et al., 2021 [12] | Hypertension disutility is applied |
| Arrhythmia | 0.0940 | 0.0094 | NA | Shiroiwa et al., 2021 [11] | Assumed to equal to palpitation  SE is assumed to be 10% of mean |
| Diabetes | 0.1442 | 0.0001 | NA | Whittington et al., 2021 [12] | - |
| Cardiomyopathy | 0.1305 | 0.0131 | NA | Shiroiwa et al., 2021 [11] | Assumed as average of breathlessness (0.159), dizziness (0.098), swelling or heaviness of legs (0.171) and palpitations (0.094) disutilities  SE is assumed to be 10% of mean |
| Sleep apnea | 0.1342 | 0.0001 | NA | Whittington et al., 2021 [12] | - |
| **Radiotherapy Utility Decrement Applied in Subsequent Treatment Health State** | | | | | |
| Radiotherapy utility decrement | 0.1172 | 0.0001 | NA | Whittington et al., 2021 [12] | Hypopituitarism disutility is applied as a proxy to subsequent treatment health state for the first 10 years of this therapy |
| *Duration of AEs impact on utilities is assumed.  Abbreviations: AE, adverse event; SE, standard errors; NA, not applicable | | | | | |

In subsequent line AE utility decrement was calculated as weighted average of disutilities of all subsequent treatment “baskets”.

# 4. Comorbidities input

Table 15. Distribution of Comorbidities for different control definitions.

| **Comorbidity** | **Full Control** | **Partial Control*** | **No Control** | **Subsequent Treatment**** |
| --- | --- | --- | --- | --- |
| **Distribution of Comorbidity (IGF-1 Biochemical Control)** | | | | |
| Moderate hypertension | 0.00% | 0.00% | 0.00% | 0.00% |
| Severe hypertension | 41.77% | 50.15% | 58.54% | 47.97% |
| Arrhythmia | 0.00% | 0.00% | 0.00% | 0.00% |
| Diabetes | 25.32% | 33.39% | 41.46% | 31.29% |
| Cardiomyopathy | 3.80% | 5.56% | 7.32% | 5.10% |
| Sleep apnea | 20.25% | 23.54% | 26.83% | 22.69% |
| **Distribution of Comorbidity (GH and IGF-1 Biochemical Control)** | | | | |
| Moderate hypertension | 13.66% | 17.32% | 20.98% | 16.59% |
| Severe hypertension | 20.98% | 25.12% | 29.27% | 24.29% |
| Arrhythmia | 1.46% | 4.15% | 6.83% | 3.61% |
| Diabetes | 12.20% | 17.56% | 22.93% | 16.49% |
| Sleep apnea | 38.89% | 47.22% | 55.56% | 45.56% |
| *Partial control distribution is assumed to be average of full control and no control ones  **In base case subsequent treatment comorbidity distribution is calculated as weighted average of full control and no control values applying 60% efficacy in subsequent line per Jenkins et al. 2006 [14]; Source: Carmichael et al., [15]  Sources: for moderate hypertension, severe hypertension, arrhythmia and diabetes is Colao et al., 2011 [16] and Davi et al., 2008 [17] for sleep apnea  Abbreviations: IGF-1, insulin-like growth factor 1; GH, growth hormone | | | | |

# 5. Cost input

Table 16. Drag acquisition costs.

| **Drug** | **Tablet/vial size** | **Number of tablets/vial**s **in pack** | **Price** | **Dose** | **Frequency per month** | **Source (Cost)** |
| --- | --- | --- | --- | --- | --- | --- |
| Pasireotide | 60 mg | 1 | R$7,050.00 | 60 mg | 1.09 | Conitec Recommendation Report N. 904/2024 [18] |
| Pasireotide | 40 mg | 1 | R$5,700.00 | 40 mg | 1.09 | Conitec Recommendation Report N. 904/2024 [18] |
| Octreotide max dose | 30 mg | 1 | R$3,444.05 | 30 mg | 1.09 | Contract N. 174/2024 (DLOG/SE/MS) [18] |
| Lanreotide max dose | 120 mg/0.5 ml | 1 | R$1,931.69 | 120 mg/0.5 ml | 1.09 | Contract N. 174/2024 (DLOG/SE/MS) [18] |
| Pegvisomant | 10 mg | 30.0 | R$8,507.11 | 10 mg | 30.44 | PMVG 18% from CMED List Prices  (Sep 11^th,^ 2025) [18] |
| Pegvisomant | 15 mg | 30.0 | R$12,760.64 | 15 mg | 30.44 | PMVG 18% from CMED List Prices  (Sep 11^th,^ 2025) [18] |
| Cabergoline | 0.5 mg | 1 | R$19.47 | 1.5 mg | 4.35 | Lowest PMVG 0% from CMED List Prices  (Sep 11^th,^ 2025) [18] |

Table 17. Drag acquisition costs per month.

| **Second-line Treatment** | **Base Dose** | **High Dose** |
| --- | --- | --- |
| Pasireotide | R$6,196 ($1,157) | R$7,664 ($1,432) |
| Octreotide max dose | R$4,992 ($932) | R$4,992 ($932) |
| Lanreotide max dose | R$2,100 ($392) | R$2,100 ($392) |
| Pegvisomant | R$11,221 ($2,096) | R$17,262 ($3,225) |
| Pegvisomant + Octreotide | R$16,691 ($3,118) | R$21,006 ($3,924) |
| Pegvisomant + Lanreotide |  |  |

Table 18. Adverse event management monthly costs.

| **Adverse Event** | **Monthly Cost** | **Source** |
| --- | --- | --- |
| Abdominal distension | R$128.62 ($24.03) | Digestive Diseases - Cost per patient 2015. |
| Abdominal pain | R$128.62 ($24.03) | Digestive Diseases - Cost per patient 2015. |
| Blood glucose increased | R$102.97 ($19.23) | Endocrine/Nutrition/Metabolic Diseases - Cost per patient 2015. |
| Cholelithiasis | R$128.62 ($24.03) | Digestive Diseases - Cost per patient 2015. |
| Decreased appetite | R$128.62 ($24.03) | Digestive Diseases - Cost per patient 2015. |
| Diarrhea | R$128.62 ($24.03) | Digestive Diseases - Cost per patient 2015. |
| Gastrointestinal disorders | R$128.62 ($24.03) | Digestive Diseases - Cost per patient 2015. |
| Headache | R$28.00 ($5.23) | 03.01.01.007-2 - CONSULTA MEDICA EM ATENÇÃO ESPECIALIZADA - 2022 Specialist Visits |
| Hyperglycemia | R$102.97 ($19.23) | Endocrine/Nutrition/Metabolic Diseases - Cost per patient 2015. |
| Infections and infestations | R$176.19 ($32.91) | Certain infectious/parasitic diseases - Cost per patient 2015. |
| Musculoskeletal and connective tissue disorders | R$218.27 ($40.77) | Musculoskeletal/connective tissue disorders - Cost per patient 2015. |
| Nausea | R$128.62 ($24.03) | Digestive Diseases - Cost per patient 2015. |
| Nervous system disorders | R$28.00 ($5.23) | 03.01.01.007-2 - CONSULTA MEDICA EM ATENÇÃO ESPECIALIZADA - 2022 Specialist Visits |
| Pain | R$28.00 ($5.23) | 03.01.01.007-2 - CONSULTA MEDICA EM ATENÇÃO ESPECIALIZADA - 2022 Specialist Visit |
| Sinus bradycardia | R$307.59 ($57.46) | Circulatory Diseases - Cost per patient 2015. |
| Tumor growth | R$3,235.89 ($604.46) | Assumed to equal to surgery cost weighted average of cost and resource use of surgery with a cost of R$2,991.07 (04.03.03.006-4 - HIPOFISECTOMIA TRANSESFENOIDAL POR TECNICA COMPLEMENTAR), MRI, pain killer and specialist visit. Weights are assumed at 92%, 50%, 30% and 70% for MRI, pain killer, surgery, and specialist visit, respectively. |
| Upper respiratory tract infection | R$139.19 ($ (26.00) | Respiratory Diseases - Cost per patient 2015. Fig 2 |
| Unit cost source: SIGTAP [19]; Values sourced from the SUS Procedures, Medications, Orthoses, Prostheses and Special Materials Table Management System (SIGTAP) were adjusted to the real value by applying the correction factor of 2.8, according to recent CONITEC assessments [20]. All other costs which were not sourced for the current year were inflated to 2025 values using the Consumer Price Index (CPI) recalculated to the inflation adjustment factor [21]. | | |

Table 19. Adverse event cost per month.

| **Adverse Event** | **Monthly Adverse Event Cost** |
| --- | --- |
| Pasireotide | R$0.97 ($0.18) |
| Octreotide max dose | R$0.17 ($0.03) |
| Lanreotide max dose | R$0.28 ($0.05) |
| Pegvisomant | R$11.20 ($2.09) |
| Pegvisomant + Octreotide | R$10.94 ($2.04) |
| Pegvisomant + Lanreotide | R$10.94 (2.04) |

Table 20. Comorbidity unit costs.

| **Comorbidity** | **Management Cost** | **Source** |
| --- | --- | --- |
| Moderate hypertension | R$307.59 | Circulatory Diseases - Cost per patient 2015. |
| Severe hypertension | R$307.59 | Circulatory Diseases - Cost per patient 2015. |
| Arrhythmia | R$307.59 | Circulatory Diseases - Cost per patient 2015. |
| Diabetes | R$102.97 | Endocrine/Nutrition/Metabolic Diseases - Cost per patient 2015. |
| Cardiomyopathy | R$307.59 | Circulatory Diseases - Cost per patient 2015. |
| Sleep apnea | R$139.19 | Respiratory Diseases - Cost per patient 2015. |
| Unit cost source: SIGTAP [19]; Values sourced from the SUS Procedures, Medications, Orthoses, Prostheses and Special Materials Table Management System (SIGTAP) were adjusted to the real value by applying the correction factor of 2.8, according to recent CONITEC assessments [20]. All other costs which were not sourced for the current year were inflated to 2025 values using the Consumer Price Index (CPI) recalculated to the inflation adjustment factor [21]. | | |

Table 21. Annual hospital stay per comorbidity.

| **Comorbidity** | **Annual hospital days** | **Source** |
| --- | --- | --- |
| Moderate hypertension | 7.27 | Zhao et al., 2020 [22] |
| Severe hypertension | 7.27 | Zhao et al., 2020 [22] |
| Arrhythmia | 7.27 | Zhao et al., 2020 [22] |
| Diabetes | 5.90 | Zhao et al., 2020 [22] |
| Cardiomyopathy | 7.27 | Zhao et al., 2020 [22] |
| Sleep apnea | 5.98 | Zhao et al., 2020 [22] |

Table 22. Comorbidity Monthly Cost per Acromegaly Control.

| **Comorbidity** | **Biochemical control: IGF-1 normalization** |
| --- | --- |
| Full Control | R$194.43 ($36.32) |
| Partial Control | R$238.52 ($44.55) |
| No Control | R$282.60 ($52.79) |
| Subsequent Treatment | R$227.05 ($42.41) |

Table 23. Calculation of Monitoring Costs.

| **Procedure** | **Unit Cost** | **PAS LAR** | **OCT** | **LAN** | **PEG** | **PEG+ OCT** | **PEG+ LAN** | **Codes** |
| --- | --- | --- | --- | --- | --- | --- | --- | --- |
| GH | R$28.59 | 1.00 | 1.00 | 1.00 | 0.00 | 1.00 | 1.00 | 02.02.06.022-5 |
| IGF-1 | R$42.98 | 1.00 | 1.00 | 1.00 | 1.00 | 1.00 | 1.00 | 02.02.06.032-2 |
| Liver panel | R$61.67 | 0.00 | 0.00 | 0.00 | 2.00 | 2.00 | 2.00 | 02.02.01.062-7; 02.02.01.020-1; 02.02.01.042-2; 02.02.01.061-9; 02.02.01.065-1; 02.02.01.064-3 |
| Fasting Plasma Glucose | R$10.16 | 2.00 | 2.00 | 2.00 | 2.00 | 2.00 | 2.00 | 02.02.01.004-0 |
| Glycated hemoglobin | R$22.01 | 2.00 | 2.00 | 2.00 | 2.00 | 2.00 | 2.00 | 02.02.01.050-3 |
| Free T4 | R$25.09 | 2.00 | 2.00 | 2.00 | 2.00 | 2.00 | 2.00 | 02.02.06.038-1 |
| Thyroid-stimulating hormone | R$25.09 | 2.00 | 2.00 | 2.00 | 2.00 | 2.00 | 2.00 | 02.02.01.025-0 |
| Consultation with endocrinologist and cardiologist | R$56.00 | 2.00 | 2.00 | 2.00 | 2.00 | 2.00 | 2.00 | 02.02.01.007-2 |
| Blood pressure measurement | R$28.20 | 2.00 | 2.00 | 2.00 | 2.00 | 2.00 | 2.00 | 02.11.02.005-2 |
| Echocardiogram | R$111.83 | 1.00 | 1.00 | 1.00 | 1.00 | 1.00 | 1.00 | 02.02.01.003-2 |
| Ecodoppler | R$327.60 | 1.00 | 1.00 | 1.00 | 1.00 | 1.00 | 1.00 | 02.02.01.001-1 |
| Electrocardiogram | R$14.42 | 1.00 | 1.00 | 1.00 | 1.00 | 1.00 | 1.00 | 02.11.02.003-6 |
| Polysomnography | R$350.00 | 1.00 | 1.00 | 1.00 | 1.00 | 1.00 | 1.00 | 02.11.05.010-5 |
| Sella turcica magnetic resonance imaging | R$752.50 | 0.00 | 0.00 | 0.00 | 1.00 | 1.00 | 1.00 | 02.02.01.007-2 |
| Total Monthly Cost | | R$101.94 ($19.04) | R$101.94 ($19.04) | R$101.94 ($19.04) | R$167.54 ($31.30) | R$169.92 ($31.74) | R$169.92 ($31.74) | Calculation |
| Note: For combination therapies PEG+OCT and PEG+LAN maximum of individual component monitoring is applied.  Unit cost source: SIGTAP [19]  Values sourced from the SUS Procedures, Medications, Orthoses, Prostheses and Special Materials Table Management System (SIGTAP) were adjusted to the real value by applying the correction factor of 2.8, according to recent CONITEC assessments [20]. All other costs which were not sourced for the current year were inflated to 2025 values using the Consumer Price Index (CPI) recalculated to the inflation adjustment factor [21].  Abbreviations: GH, growth hormone; IGF-1, insulin-like growth factor-1; LAN, lanreotide; OCT, Octreotide; PAS LAR, pasireotide long-acting release; PEG, pegvisomant; | | | | | | | | |

Table 24. Subsequent treatment costs.

| **Subsequent Treatment Costs Category** | **Monthly/one time cost** | **Source** |
| --- | --- | --- |
| Treatment costs* | R$4,133 ($772) | Contract N. 174/2024 (DLOG/SE/MS) |
| Radiotherapy costs** | R$11,670 ($2,180) | SIGTAP Procedure 03.04.01-050-2 (http://sigtap.datasus.gov.br/) |
| * Monthly treatment costs are based on a weighted distribution of maximum doses of octreotide and lanreotide, with a 70.3% and 29.7% split, respectively.  ** One time radiotherapy cost is applied upon transition from second line therapy to subsequent treatment.  Values sourced from the SUS Procedures, Medications, Orthoses, Prostheses and Special Materials Table Management System (SIGTAP) were adjusted to the real value by applying the correction factor of 2.8, according to recent CONITEC assessments [20]. All other costs which were not sourced for the current year were inflated to 2025 values using the Consumer Price Index (CPI) recalculated to the inflation adjustment factor [21]. | | |

# References

[1]	Biagetti B, Araujo-Castro M, Tebe C, et al. Real-world evidence of effectiveness and safety of pasireotide in the treatment of acromegaly: a systematic review and meta-analysis. Rev Endocr Metab Disord 2025;26:97–111. https://doi.org/10.1007/s11154-024-09928-3.

[2]	Leonart LP, Tonin FS, Ferreira VL, et al. Effectiveness and safety of pegvisomant: a systematic review and meta-analysis of observational longitudinal studies. Endocrine 2019;63:18–26. https://doi.org/10.1007/s12020-018-1729-7.

[3]	Urbani C, Dassie F, Zampetti B, et al. Real-life data of Pasireotide LAR in acromegaly: a long-term follow-up. J Endocrinol Invest 2024;47:1733–41. https://doi.org/10.1007/s40618-023-02275-1.

[4]	Gadelha MR, Bronstein MD, Brue T, et al. Pasireotide versus continued treatment with octreotide or lanreotide in patients with inadequately controlled acromegaly (PAOLA): a randomised, phase 3 trial. Lancet Diabetes Endocrinol 2014;2:875–84. https://doi.org/10.1016/S2213-8587(14)70169-X.

[5]	Trainer PJ, Drake WM, Katznelson L, et al. Treatment of acromegaly with the growth hormone-receptor antagonist pegvisomant. N Engl J Med 2000;342:1171–7. https://doi.org/10.1056/NEJM200004203421604.

[6]	Colao A, Zgliczynski W, Komorowski J, et al. Efficacy and safety of high-dose long-acting repeatable octreotide as monotherapy or in combination with pegvisomant or cabergoline in patients with acromegaly not adequately controlled by conventional regimens: results of an open-label, multicentre study. Endokrynol Pol 2019;70:305–12. https://doi.org/10.5603/EP.a2019.0023.

[7]	Garcia Basavilbaso N, Guitelman M, Nagelberg A, et al. Experience from the Argentine Pegvisomant Observational Study: preliminary data. Front Horm Res 2010;38:42–9. https://doi.org/10.1159/000318493.

[8]	Haliloglu O., Kuruoglu E., Ozkaya H.M., et al. Multidisciplinary Approach for Acromegaly: A Single Tertiary Center’s Experience. World Neurosurg 2016;88:270 EP – 276. https://doi.org/10.1016/j.wneu.2015.12.092.

[9]	Trainer PJ, Ezzat S, D’Souza GA, et al. A randomized, controlled, multicentre trial comparing pegvisomant alone with combination therapy of pegvisomant and long-acting octreotide in patients with acromegaly. Clin Endocrinol (Oxf) 2009;71:549–57. https://doi.org/10.1111/j.1365-2265.2009.03620.x.

[10]	Andries M, Glintborg D, Kvistborg A, et al. A 12-month randomized crossover study on the effects of lanreotide Autogel and octreotide long-acting repeatable on GH and IGF-l in patients with acromegaly. Clin Endocrinol Oxf 2008;68:473–80. https://doi.org/10.1111/j.1365-2265.2007.03067.x.

[11]	Shiroiwa T, Noto S, Fukuda T. Japanese Population Norms of EQ-5D-5L and Health Utilities Index Mark 3: Disutility Catalog by Disease and Symptom in Community Settings. Value Health J Int Soc Pharmacoeconomics Outcomes Res 2021;24:1193–202. https://doi.org/10.1016/j.jval.2021.03.010.

[12]	Whittington MD, Munoz KA, Whalen JD, et al. Economic and clinical burden of comorbidities among patients with acromegaly. Growth Horm IGF Res Off J Growth Horm Res Soc Int IGF Res Soc 2021;59:101389. https://doi.org/10.1016/j.ghir.2021.101389.

[13]	Harris S, Mamdani M, Galbo-Jørgensen CB, et al. The effect of hypoglycemia on health-related quality of life: Canadian results from a multinational time trade-off survey. Can J Diabetes 2014;38:45–52. https://doi.org/10.1016/j.jcjd.2013.09.001.

[14]	Jenkins PJ, Bates P, Carson MN, et al. Conventional pituitary irradiation is effective in lowering serum growth hormone and insulin-like growth factor-I in patients with acromegaly. J Clin Endocrinol Metab 2006;91:1239–45. https://doi.org/10.1210/jc.2005-1616.

[15]	Carmichael JD, Broder MS, Cherepanov D, et al. The association between biochemical control and cardiovascular risk factors in acromegaly. BMC Endocr Disord 2017;17:15. https://doi.org/10.1186/s12902-017-0166-6.

[16]	Colao A, Pivonello R, Grasso LFS, et al. Determinants of cardiac disease in newly diagnosed patients with acromegaly: results of a 10 year survey study. Eur J Endocrinol 2011;165:713–21. https://doi.org/10.1530/EJE-11-0408.

[17]	Davi’ MV, Dalle Carbonare L, Giustina A, et al. Sleep apnoea syndrome is highly prevalent in acromegaly and only partially reversible after biochemical control of the disease. Eur J Endocrinol 2008;159:533–40. https://doi.org/10.1530/EJE-08-0442.

[18]	Agência Nacional de Vigilância Sanitária (ANVISA). Câmara de Regulação do Mercado de Medicamentos (CMED) – Preços de medicamentos 2025. https://www.gov.br/anvisa/pt-br/assuntos/medicamentos/cmed/precos (accessed October 31, 2025).

[19]	Ministério da Saúde. Sistema de Gerenciamento da Tabela de Procedimentos, Medicamentos e OPM do SUS (SIGTAP). http://sigtap.datasus.gov.br/tabela-unificada/app/sec/inicio.jsp. Accessed 9 May 2025. n.d.

[20]	BRASIL M da S. Contas do SUS na perspectiva da contabilidade internacional: Brasil, 2010-2014 [Internet]. 1o ed. Fundação Oswaldo Cruz; 2018. Available in <www.saude.gov.br/bvs> n.d.

[21]	Instituto Brasileiro de Geografia e Estatística (IBGE). National Consumer Price Index (IPCA). https://www.ibge.gov.br/en/statistics/economic/prices-and-costs/17136-national-consumer-pricce-index.html. Accessed 9 May 2025. n.d.

[22]	Zhao Q, Coelho MSZS, Li S, et al. Trends in Hospital Admission Rates and Associated Direct Healthcare Costs in Brazil: A Nationwide Retrospective Study between 2000 and 2015. Innov Camb Mass 2020;1:100013. https://doi.org/10.1016/j.xinn.2020.04.013.
