# Supplementary material for: Cost−effectiveness of pasireotide as second−line therapy for adult acromegaly patients from the perspective of the Brazilian Unified Health System
Source: Front Endocrinol (Lausanne). 2026 Aug 5;17:1771580. doi: 10.3389/fendo.2026.1771580 (PMC13487751; doi:10.3389/fendo.2026.1771580)
Supplement: Supplementary file 4 [file SupplementaryFile4.docx]

**Table 25: Scenario analysis results for incremental QALY, incremental costs (US$), incremental cost-effectiveness ration (US$/QALY) and incremental net monetary benefit (US$).**

| **Scenario** | **PAS LAR vs.** | **OCT** | **LAN** | **PEG** | **PEG+OCT** | **PEG+LAN** |
| --- | --- | --- | --- | --- | --- | --- |
| **Base Case** | **Incremental QALYs** | 1.2172 | 1.2172 | 0.1683 | -0.0358 | 0.0992 |
|  | **Incremental Costs** | R$196,470 (US$36,701) | R$205,146 (US$38,321) | -R$538,945 (-US$100,675) | -R$979,624 (-US$182,994) | -R$782,542 (-US$146,179) |
|  | **ICER (per QALY) gained** | R$161,411 (US$30,152) | R$168,538 (US$31,483) | PAS is dominant | PAS is less costly, less effective | PAS is dominant |
|  | **INMB** | -R$50,406 (-US$9,416) | -R$59,081 (-US$11,036) | R$559,138 (US$104,447) | R$975,333 (US$182,192) | R$794,443 (US$148,402) |
| 1. Alternative efficacy source for comparison with pegvisomant regimens – RWE scenario | Incremental QALYs | NA | NA | 0.1064 | 0.1064 | 0.1064 |
|  | Incremental Costs | NA | NA | -R$1,588,165 (-US$296,669) | -R$1,588,165 (-US$296,669) | -R$1,588,165 (-US$296,669) |
|  | ICER (per QALY) gained | NA | NA | PAS is dominant | PAS is dominant | PAS is dominant |
|  | INMB | NA | NA | R$1,600,930 (US$299,053) | R$1,600,930 (US$299,053) | R$1,600,930 (US$299,053) |
| 2. PAS LAR Efficacy source based on C2305 study | Incremental QALYs | 1.0184 | 1.0906 | 0.1608 | -0.0652 | 0.0926 |
|  | Incremental Costs | R$181,554 (US$33,914) | R$229,747 (US$42,917) | -R$626,763 (-US$117,079) | -R$1134,893 (-US$211,998) | -R$907,792 (-US$169,576) |
|  | ICER (per QALY) gained | R$178,275 (US$33,302) | R$210,668 (US$39,353) | PAS is dominant | PAS is less costly, less effective | PAS is dominant |
|  | INMB | -R$59,347 (-US$11,086) | -R$98,879 (-US$18,471) | R$646,062 (US$120,684) | R$1127,074 (US$210,537) | R$918,910 (US$171,652) |
| 3. PAS LAR efficacy source for comparison with FGSRLs based on C2413 study with biochemical control criteria GH<1 μg/l and normalized IGF-1 | Incremental QALYs | 0.5077 | 0.5077 | NA | NA | NA |
|  | Incremental Costs | R$83,095 (US$15,522) | R$91,771 (US$17,143) | NA | NA | NA |
|  | ICER (per QALY) gained | R$163,675 (US$30,574) | R$180,761 (US$33,766) | NA | NA | NA |
|  | INMB | -R$22,173 (-US$4,142) | -R$30,848 (-US$5,762) | NA | NA | NA |
| 4. Alternative biochemical control criteria for comparison with FGSRLs: GH <1 µg/L and normalized IGF-1 | Incremental QALYs | 0.9364 | 0.9364 | NA | NA | NA |
|  | Incremental Costs | R$156,471 (US$29,229) | R$165,147 (US$30,849) | NA | NA | NA |
|  | ICER (per QALY) gained | R$167,099 (US$31,214) | R$176,363 (US$32,945) | NA | NA | NA |
|  | INMB | -R$44,104 (-US$8,239) | -R$52,778 (-US$9,859) | NA | NA | NA |
| 5. Partial control patients continue treatment beyond month 6 | Incremental QALYs | 1.5927 | 1.5927 | 0.1908 | -0.0151 | 0.1584 |
|  | Incremental Costs | R$332,692 ($62,147) | R$341,368 ($63,767) | R$-916,289 ($-171,163) | R$-1504,031 ($-280,953) | R$-1196,112 ($-223,434) |
|  | ICER (per QALY) gained | R$208,890 ($39,021) | R$214,336 ($40,038) | PAS is dominant | PAS is less costly, less effective | PAS is dominant |
|  | INMB | R$-141,572 ($-26,446) | R$-150,246 ($-28,066) | R$939,180 ($175,439) | R$1502,224 ($280,616) | R$1215,120 ($226,984) |
| 6. Treatment effect loss assumed from year 1 | Incremental QALYs | 1.2172 | 1.2172 | 0.3789 | 0.2152 | 0.3230 |
|  | Incremental Costs | R$196,470 ($36,701) | R$205,146 ($38,321) | R$-390,364 ($-72,920) | R$-752,428 ($-140,554) | R$-592,649 ($-110,707) |
|  | ICER (per QALY) gained | R$161,411 ($30,152) | R$168,538 ($31,483) | PAS is dominant | PAS is dominant | PAS is dominant |
|  | INMB | R$-50,406 ($-9,416) | R$-59,081 ($-11,036) | R$435,834 ($81,414) | R$778,252 ($145,377) | R$631,410 ($117,947) |
| 7. Fixed PEG dose over lifetime | Incremental QALYs | 1.2172 | 1.2172 | 0.1683 | -0.0358 | 0.0992 |
|  | Incremental Costs | R$196 470 (US$36,701) | R$205 146 (US$38,321) | -R$338 229 (-US$63,181) | -R$597 288 (-US$111,573) | -R$441 528 (-US$82,477) |
|  | ICER (per QALY) gained | R$161 411 (US$30,152) | R$168 538 (US$31,483) | PAS is dominant | PAS is less costly, less effective | PAS is dominant |
|  | INMB | -R$50 406 (-US$9,416) | -R$59 081 (US$-US$11,036) | R$358 422 (US$66,953) | R$592 997 (US$110,772) | R$453 428 (US$84,700) |
| 8. Ceiling PEG dose based on RWE data | Incremental QALYs | 1.2172 | 1.2172 | 0.1683 | -0.0358 | 0.0992 |
|  | Incremental Costs | R$196,470 ($36,701) | R$205,146 ($38,321) | R$-369,083 ($-68,945) | R$-643,984 ($-120,296) | R$-483,178 ($-90,258) |
|  | ICER (per QALY) gained | R$161,411 ($30,152) | R$168,538 ($31,483) | PAS is dominant | PAS is less costly, less effective | PAS is dominant |
|  | INMB | R$-50,406 ($-9,416) | R$-59,081 ($-11,036) | R$389,276 ($72,717) | R$639,693 ($119,495) | R$495,078 ($92,481) |
| 9. Addition of FGSRLs to PEG monotherapy with ceiling PEG dose based on RWE | Incremental QALYs | 1.2172 | 1.2172 | 0.1683 | -0.0358 | 0.0992 |
|  | Incremental Costs | R$196,470 ($36,701) | R$205,146 ($38,321) | R$-453,277 ($-84,672) | R$-643,984 ($-120,296) | R$-483,178 ($-90,258) |
|  | ICER (per QALY) gained | R$161,411 ($30,152) | R$168,538 ($31,483) | PAS is dominant | PAS is less costly, less effective | PAS is dominant |
|  | INMB | R$-50,406 ($-9,416) | R$-59,081 ($-11,036) | R$473,470 ($88,444) | R$639,693 ($119,495) | R$495,078 ($92,481) |
| 10. PEG prices without VAT | Incremental QALYs | NA | NA | 0.1683 | -0.0358 | 0.0992 |
|  | Incremental Costs | NA | NA | -R$386,025 (-US$72,110) | -R$782,811 (-US$146,229) | -R$601,294 (-US$112,322) |
|  | ICER (per QALY) gained | NA | NA | PAS is dominant | PAS is less costly, less effective | PAS is dominant |
|  | INMB | NA | NA | R$406,218 (US$75,882) | R$778,520 (US$145,428) | R$613,195 (US$114,545) |
| 11. PEG lower-bound price | Incremental QALYs | 1.2172 | 1.2172 | 0.1683 | -0.0358 | 0.0992 |
|  | Incremental Costs | R$196,470 ($36,701) | R$205,146 ($38,321) | R$-417,151 ($-77,924) | R$-877,220 ($-163,865) | R$-691,088 ($-129,095) |
|  | ICER (per QALY) gained | R$161,411 ($30,152) | R$168,538 ($31,483) | PAS is dominant | PAS is less costly, less effective | PAS is dominant |
|  | INMB | R$-50,406 ($-9,416) | R$-59,081 ($-11,036) | R$437,344 ($81,696) | R$872,929 ($163,063) | R$702,988 ($131,318) |
| 12. PEG upper-bound price | Incremental QALYs | 1.2172 | 1.2172 | 0.1683 | -0.0358 | 0.0992 |
|  | Incremental Costs | R$196,470 ($36,701) | R$205,146 ($38,321) | R$-591,632 ($-110,517) | R$-1050,327 ($-196,201) | R$-845,887 ($-158,012) |
|  | ICER (per QALY) gained | R$161,411 ($30,152) | R$168,538 ($31,483) | PAS is dominant | PAS is less costly, less effective | PAS is dominant |
|  | INMB | R$-50,406 ($-9,416) | R$-59,081 ($-11,036) | R$611,825 ($114,289) | R$1046,036 ($195,399) | R$857,787 ($160,235) |
| 13. PEG hypothetical price if reimbursed | Incremental QALYs | 1.2172 | 1.2172 | 0.1683 | -0.0358 | 0.0992 |
|  | Incremental Costs | R$196,470 ($36,701) | R$205,146 ($38,321) | R$-46,281 ($-8,645) | R$-332,513 ($-62,114) | R$-202,594 ($-37,845) |
|  | ICER (per QALY) gained | R$161,411 ($30,152) | R$168,538 ($31,483) | PAS is dominant | PAS is less costly, less effective | PAS is dominant |
|  | INMB | R$-50,406 ($-9,416) | R$-59,081 ($-11,036) | R$66,474 ($12,417) | R$328,222 ($61,312) | R$214,494 ($40,068) |
| 14. Chronic PAS LAR-related hyperglycemia management | Incremental QALYs | 1.2172 | 1.2172 | 0.1683 | -0.0358 | 0.0992 |
|  | Incremental Costs | R$198,487 ($37,077) | R$207,163 ($38,698) | R$-536,928 ($-100,298) | R$-977,607 ($-182,617) | R$-780,526 ($-145,802) |
|  | ICER (per QALY) gained | R$163,068 ($30,461) | R$170,194 ($31,792) | PAS is dominant | PAS is less costly, less effective | PAS is dominant |
|  | INMB | R$-52,422 ($-9,793) | R$-61,097 ($-11,413) | R$557,121 ($104,070) | R$973,316 ($181,815) | R$792,426 ($148,025) |
| 15. Delayed RT effect and adjusted RT outcome | Incremental QALYs | 1.3486 | 1.3487 | 0.1853 | -0.0404 | 0.1090 |
|  | Incremental Costs | R$163,279 (US$30,501) | R$171,955 (US$32,121) | -R$543,236 (-US$101,477) | -R$978,459 (-US$182,776) | -R$785,024 (-US$146,642) |
|  | ICER (per QALY) gained | R$121,069 (US$22,616) | R$127,501 (US$23,817) | PAS is dominant | PAS is less costly, less effective | PAS is dominant |
|  | INMB | -R$1,441 (-US$,269) | -R$10,116 (-US$1,890) | R$565,471 (US$105,630) | R$973,616 (US$181,871) | R$798,109 (US$149,087) |
| 16. Alternative source for health state utility values, Rowles et al. (2005) | Incremental QALYs | 1.4478 | 1.4478 | 0.1975 | -0.0436 | 0.1168 |
|  | Incremental Costs | R$196,470 (US$36,701) | R$205,146 (US$38,321) | -R$538,945 (-US$100,675) | -R$979,624 (-US$182,994) | -R$782,542 (-US$146,179) |
|  | ICER (per QALY) gained | R$135,702 (US$25,349) | R$141,693 (US$26,468) | PAS is dominant | PAS is less costly, less effective | PAS is dominant |
|  | INMB | -R$22,733 (-US$4,246) | -R$31,408 (-US$5,867) | R$562,645 (US$105,102) | R$974,390 (US$182,016) | R$796,557 (US$148,797) |
| 17. Exclusion of disutilities for AEs | Incremental QALYs | 1.2183 | 1.2183 | 0.1605 | -0.0422 | 0.0935 |
|  | Incremental Costs | R$196,470 (US$36,701) | R$205,146 (US$38,321) | -R$538,945 (-US$100,675) | -R$979,624 (-US$182,994) | -R$782,542 (-US$146,179) |
|  | ICER (per QALY) gained | R$161,271 (US$30,125) | R$168,392 (US$31,456) | PAS is dominant | PAS is less costly, less effective | PAS is dominant |
|  | INMB | -R$50,279 (-US$9,392) | -R$58,954 (-US$11,013) | R$558,206 (US$104,273) | R$974,558 (US$182,047) | R$793,759 (US$148,274) |
| 18. Exclusion of disutilities for comorbidities | Incremental QALYs | 1.1900 | 1.1900 | 0.1644 | -0.0348 | 0.0971 |
|  | Incremental Costs | R$196,470 (US$36,701) | R$205,146 (US$38,321) | -R$538,945 (-US$100,675) | -R$979,624 (-US$182,994) | -R$782,542 (-US$146,179) |
|  | ICER (per QALY) gained | R$165,105 (US$30,842) | R$172,395 (US$32,203) | PAS is dominant | PAS is less costly, less effective | PAS is dominant |
|  | INMB | -R$53,674 (-US$10,026) | -R$62,348 (-US$11,647) | R$558,675 (US$104,361) | R$975,453 (US$182,215) | R$794,198 (US$148,356) |
| 19. Partial control comorbidities closer to full control comorbidities | Incremental QALYs | 1.2177 | 1.2177 | 0.1681 | -0.0357 | 0.0992 |
|  | Incremental Costs | R$196,462 ($36,699) | R$205,138 ($38,320) | R$-538,942 ($-100,674) | R$-979,624 ($-182,994) | R$-782,543 ($-146,179) |
|  | ICER (per QALY) gained | R$161,344 ($30,139) | R$168,468 ($31,470) | PAS is dominant | PAS is less costly, less effective | PAS is dominant |
|  | INMB | R$-50,343 ($-9,404) | R$-59,018 ($-11,025) | R$559,119 ($104,443) | R$975,334 ($182,192) | R$794,448 ($148,403) |
| 20. Proportion of PEG AEs being severe set as 50% of PAS LAR | Incremental QALYs | 1.2172 | 1.2172 | 0.1640 | -0.0358 | 0.0992 |
|  | Incremental Costs | R$196,470 ($36,701) | R$205,146 ($38,321) | R$-538,829 ($-100,653) | R$-979,624 ($-182,994) | R$-782,542 ($-146,179) |
|  | ICER (per QALY) gained | R$161,411 ($30,152) | R$168,538 ($31,483) | PAS is dominant | PAS is less costly, less effective | PAS is dominant |
|  | INMB | R$-50,406 ($-9,416) | R$-59,081 ($-11,036) | R$558,509 ($104,330) | R$975,333 ($182,192) | R$794,443 ($148,402) |
| 21. Proportion of PEG AEs being severe set as 200% of PAS LAR | Incremental QALYs | 1.2172 | 1.2172 | 0.1770 | -0.0358 | 0.0992 |
|  | Incremental Costs | R$196,470 ($36,701) | R$205,146 ($38,321) | R$-539,180 ($-100,719) | R$-979,624 ($-182,994) | R$-782,542 ($-146,179) |
|  | ICER (per QALY) gained | R$161,411 ($30,152) | R$168,538 ($31,483) | PAS is dominant | PAS is less costly, less effective | PAS is dominant |
|  | INMB | R$-50,406 ($-9,416) | R$-59,081 ($-11,036) | R$560,414 ($104,685) | R$975,333 ($182,192) | R$794,443 ($148,402) |
| 22. Time horizon 20 years | Incremental QALYs | 0.9514 | 0.9514 | 0.1321 | -0.0279 | 0.0774 |
|  | Incremental Costs | R$141,209 (US$26,378) | R$149,885 (US$27,999) | -R$396,181 (-US$74,007) | -R$759,732 (-US$141,918) | -R$606,398 (-US$113,275) |
|  | ICER (per QALY) gained | R$148,425 (US$27,726) | R$157,543 (US$29,429) | PAS is dominant | PAS is less costly, less effective | PAS is dominant |
|  | INMB | -R$27,043 (-US$5,052) | -R$35,718 (-US$6,672) | R$412,034 (US$76,968) | R$756,382 (US$141,292) | R$615,692 (US$115,011) |
| 23. Time horizon 5 years | Incremental QALYs | 0.3836 | 0.3836 | 0.0541 | -0.0118 | 0.0300 |
|  | Incremental Costs | R$39,100 (US$7,304) | R$47,775 (US$8,924) | -R$122,942 (-US$22,965) | -R$233,712 (-US$43,657) | -R$182,845 (-US$34,155) |
|  | ICER (per QALY) gained | R$101,927 (US$19,040) | R$124,541 (US$23,264) | PAS is dominant | PAS is less costly, less effective | PAS is dominant |
|  | INMB | R$6,933 (US$1,295) | -R$1,742 (-US$,325) | R$129,435 (US$24,178) | R$232,296 (US$43,393) | R$186,446 (US$34,828) |
| 24. Perspective, societal | Incremental QALYs | 1.2172 | 1.2172 | 0.1683 | -0.0358 | 0.0992 |
|  | Incremental Costs | R$196,412 (US$36,690) | R$205,087 (US$38,310) | -R$538,953 (-US$100,676) | -R$979,622 (-US$182,993) | -R$782,547 (-US$146,180) |
|  | ICER (per QALY) gained | R$161,363 (US$30,143) | R$168,489 (US$31,474) | PAS is dominant | PAS is less costly, less effective | PAS is dominant |
|  | INMB | -R$50,347 (-US$9,405) | -R$59,022 (-US$11,025) | R$559,146 (US$104,448) | R$975,330 (US$182,192) | R$794,447 (US$148,403) |
| 25. Discount Rate 0% | Incremental QALYs | 2.5074 | 2.5074 | 0.3443 | -0.0735 | 0.2052 |
|  | Incremental Costs | R$452,946 (US$84,610) | R$461,622 (US$86,231) | -R$1197,498 (-US$223,693) | -R$2066,248 (-US$385,975) | -R$1653,532 (-US$308,880) |
|  | ICER (per QALY) gained | R$180,642 (US$33,744) | R$184,101 (US$34,390) | PAS is dominant | PAS is less costly, less effective | PAS is dominant |
|  | INMB | -R$152,055 (-US$28,404) | -R$160,730 (-US$30,024) | R$1238,810 (US$231,410) | R$2057,430 (US$384,328) | R$1678,159 (US$313,480) |
| 26. Discount Rate 1.5% | Incremental QALYs | 1.9521 | 1.9521 | 0.2686 | -0.0572 | 0.1596 |
|  | Incremental Costs | R$342,378 (US$63,956) | R$351,054 (US$65,577) | -R$914,577 (-US$170,843) | -R$1604,814 (-US$299,779) | -R$1283,722 (-US$239,799) |
|  | ICER (per QALY) gained | R$175,388 (US$32,763) | R$179,832 (US$33,593) | PAS is dominant | PAS is less costly, less effective | PAS is dominant |
|  | INMB | -R$108,125 (-US$20,198) | -R$116,799 (-US$21,818) | R$946,808 (US$176,864) | R$1597,951 (US$298,497) | R$1302,880 (US$243,378) |
| 27. Discount Rate 10% | Incremental QALYs | 0.7596 | 0.7596 | 0.1057 | -0.0225 | 0.0614 |
|  | Incremental Costs | R$108,377 (US$20,245) | R$117,052 (US$21,865) | -R$309,381 (-US$57,792) | -R$582,708 (-US$108,850) | -R$464,318 (-US$86,735) |
|  | ICER (per QALY) gained | R$142,671 (US$26,651) | R$154,090 (US$28,784) | PAS is dominant | PAS is less costly, less effective | PAS is dominant |
|  | INMB | -R$17,222 (-US$3,217) | -R$25,896 (-US$4,837) | R$322,062 (US$60,161) | R$580,005 (US$108,345) | R$471,682 (US$88,110) |
