# Supplementary material for: Cost−effectiveness of pasireotide as second−line therapy for adult acromegaly patients from the perspective of the Brazilian Unified Health System
Source: Front Endocrinol (Lausanne). 2026 Aug 5;17:1771580. doi: 10.3389/fendo.2026.1771580 (PMC13487751; doi:10.3389/fendo.2026.1771580)
Supplement: Supplementary file 5 [file SupplementaryFile5.docx]

**Figures 3 – 7:** **DSA results for incremental QALY and incremental costs (US$).**

**Figure 3. PAS LAR vs OCT**

**Figure 4. PAS LAR vs LAN**

**Figure 5. PAS LAR vs PEG**

**Figure 6. PAS LAR vs PEG+OCT**

**Figure 7. PAS LAR vs PEG+LAN**

Abbreviations: QALY, Quality adjusted life years; NC, No control; FC, Full control; PC, Partial control; AE, Adverse event; SMR, Standardized mortality ratio
