# Supplementary material for: Cost−effectiveness of pasireotide as second−line therapy for adult acromegaly patients from the perspective of the Brazilian Unified Health System
Source: Front Endocrinol (Lausanne). 2026 Aug 5;17:1771580. doi: 10.3389/fendo.2026.1771580 (PMC13487751; doi:10.3389/fendo.2026.1771580)
Supplement: Supplementary file 6 [file SupplementaryFile6.docx]

**Figures 8- 12: PSA results, cost-effectiveness acceptability curves**

**Fig 8. PAS LAR vs OCT**

Abbreviations: QALY, Quality adjusted life years

**Fig 9. PAS LAR vs LAN**

Abbreviations: QALY, Quality adjusted life years

**Fig 10. PAS LAR vs PEG**

Abbreviations: QALY, Quality adjusted life years

**Fig 11. PAS LAR vs PEG+OCT**

Abbreviations: QALY, Quality adjusted life years

**Fig 12. PAS LAR vs PEG+LAN**

Abbreviations: QALY, Quality adjusted life years
